# Exploration of Food Security Challenges towards More Sustainable Food Production: A Systematic Literature Review of the Major Drivers and Policies

**DOI:** 10.3390/foods11233804

**Published:** 2022-11-25

**Authors:** Sabreen Wahbeh, Foivos Anastasiadis, Balan Sundarakani, Ioannis Manikas

**Affiliations:** 1 Faculty of Business, University of Wollongong in Dubai, Dubai 20183, United Arab Emirates; 2 Department of Agribusiness and Supply Chain Management, Agricultural University of Athens, 11855 Athens, Greece

**Keywords:** sustainable food supply chains, agri-food sector, policy recommendation, sustainability, food security

## Abstract

Food security is a central priority for international policy as one of the world’s most significantly urgent targets to achieve. It is considered one of the most pressing issues in many countries, the degree of food security representing the level of self-sufficiency and well-being of citizens. In particular, in the current COVID-19 pandemic era, it has more than ever become a mission-critical goal. In this research, we report on the food security drivers and the current state of recommended policies addressing chronic food insecurity aimed at ensuring the sustainability of future food production. Mapping the determinants of food security contributes to a better understanding of the issue and aids in the development of appropriate food security policies and strategies to enhance the sustainability of food production in all facets; namely environmental, social, and economic. Adopting the Preferred Reporting Items for Systematic Reviews and Meta-Analyses (PRISMA) data screening and selection guidelines and standards, we carried out a comprehensive, reliable, systematic, and rigorous review of research from the last ten years in order to identify the most frequently mentioned drivers and policies of food security in the literature available in two databases: Scopus and Web of Science (WOS). The number of extracted articles was 141 papers in total. An analysis revealed 34 drivers of food security and 17 most recommended policies for the mitigation of food insecurity. The existence of food loss and waste (FLW) policies was the primary driver of food security, followed by food security policies (FSP) in their different forms. However, FSP were the most recommended policies, followed by FLW policies. The identified food security drivers and recommended policies should be used by policy-makers to improve food security, thus contributing to sustainable food production. Our research findings, reflected in the latest version of the Global Food Security Index (GFSI), resulted in more tangible policy implications, suggesting the addition of two dimensions regarding food security. We also identified elements not listed under the GFSI that could be considered in its future revision, including environmental policies/indicators, consumer representation, and traceability throughout the entire supply chain. Overall, it can be concluded that food security is a complicated and multi-faceted issue that cannot be restricted to a single variable, necessitating the deeper integration of various multi-disciplinary interventions.

## 1. Introduction

Food security (FS) is “a situation that exists when all people, at all times, have physical, social and economic access to sufficient, safe and nutritious food that meets their dietary needs and food preferences for an active and healthy life” [1] p.3. It is a significant priority for international policy [2], and has been perceived as being among the key challenges worldwide [3] as it represents a country’s degree of self-sufficiency and the well-being of its citizens [4]. Securing a nation’s self-sufficiency has become a top priority in the context of the current COVID-19 global epidemic era, even more so than earlier [5]. Economic expansion, rising incomes, urbanization, and growing population are driving up the demand for food, as people adopt more diverse and resource-intensive dietary habits [2,6]. The world’s current population is steadily increasing, placing significant pressure on the available natural resources to feed the growing population [7,8,9]; however, this dramatic growth in the global population is anticipated mainly in developing countries, which already suffer from devastating hunger and food insecurity [7]. One of the biggest obstacles to ensuring global food security is the need to roughly double food production within the coming few decades, particularly in the context of the developing world’s rapidly increasing demand [10,11]. The natural resources such as land, water, energy, and other resources used in food production are all subject to increasing competition [12,13]. Climate change poses difficulties for agricultural production [14], mainly in developing nations, while some existing farming practices harm the environment and contribute significantly to greenhouse gas emissions (GHG) [15,16]. There is a real danger that less developed countries may be forced to reverse direction. The FAO’s statistics on world hunger in 2009 showed a dramatic rise to 1.023 billion people, demonstrating precisely such a situation. When commodity prices fell the following year, this number dropped to 925 million, which was still more prominent than in 2007 (i.e., before the price spike) [17]. According to recent data published by the Global Hunger Index, the number of malnourished people grew from 785 million in 2015 to 822 million in 2018. Moreover, 43 out of 117 countries reported extreme hunger [18]. Approximately 20% of developing countries lack the resources and physical access necessary to provide their citizens with the most basic food. Children in developing countries face vitamin and nutritional deficiencies and being underweight, which puts them at risk for various sicknesses due to food insecurity [12]. National and global imbalances brought on by food insecurity are expected to worsen human suffering and make it harder for people to survive [12]. Despite the efforts of multiple global organizations such as the FAO and the UN, the problem of food insecurity is worsening [19], which means that more effective and sustainable solutions must be provided to ensure the alleviation of food insecurity and the sustainability of food production. Hence, policy-makers must understand that in a world that is becoming more globalized, food insecurity in one region could have significant political, economic, and environmental impacts elsewhere [2].

Throughout the twentieth century, policy-makers used the concept of food security as a key notion in formulating food-related policies [17]. Lang and Barling [17] have proposed two main schools of thought on food security: the first focused on increased production as the primary solution to under-consumption and hunger, while the second is a newer one that is more socially and environmentally conscious and accepts the need to address a wide range of issues, not just production. The former is primarily concerned with agriculture, while the latter is concerned with food systems. One approach to solve the food security challenge is to intensify agricultural production in ways that impose much less environmental stress and do not jeopardize our long-term ability to continue producing food [2]. The above sustainable intensification strategy comprises a policy agenda for several governments worldwide, but has also drawn criticism for being overly production-focused or incoherent [2]. The central mission of the twenty-first century is to establish a sustainable food system, which calls for a more concrete policy framework than that which is currently in place [17]. This mission has been disrupted by competing solutions for policy focus and policies that have, so far, failed to incorporate the complex array of evidence from social, environmental, and economic components into such an integrated and comprehensive policy response [17]. Millions of people are being pushed into a cycle of food insecurity and poverty due to climate change; however, we can combat both food insecurity and climate change by implementing climate-friendly agricultural production methods [12]. Tsolakis and Srai [20] have stated that any comprehensive food security policy should entail multi-dimensional policies considering aspects such as resilience, trade, self-sufficiency, food waste, and sustainability. As it is traditionally understood, food security concerns individuals, while ecological and environmental concepts operate locally and at supra-national, regional, and international levels [1]. According to Guiné, Pato [21], the four pillars of food security—availability, access, utilization, and stability—should be reconsidered to include additional factors such as climate change. Clapp, Moseley [22] has also stressed that it is time to officially update the existing food security definition to involve two further dimensions—sustainability and agency—containing broader dynamics that have an impact on hunger and malnutrition [23]. Sustainability relates to the long-term ability of food systems to ensure food and nutrition security in a way that does not jeopardize the economic, social, and environmental foundations that generate food and nutrition security for upcoming generations [22,23]. Agency represents the ability of people or groups to decide what they consume, what they produce, and how they produce, process, and distribute their food within food systems, as well as their capacity to participate in processes that shape the food system’s policies and governance [22,23]. Instead of dismissing food security as being insufficient, Clapp, Moseley [22] has contended that the inclusion of two extra dimensions—agency and sustainability—into food security policy and assessment frameworks will help to guarantee that every human has access to food, not just now but also in the future. Sustainability can be viewed as a pre-requisite for long-term food security [1]. Environmental aspects—particularly climate and the availability of natural resources—are pre-requisite for food availability and biodiversity protection [24]. The availability of food for everybody depends on economic and social sustainability. Food utilization, too, is influenced by social sustainability. The three components of sustainability—social, economic, and environmental—ensure the continuity of the three food security dimensions and the food system stability on which they rely. As confirmation of the vital relationship between food security and sustainability, “The International Food Policy Research Institute” has launched a 2020 Vision of Food Security to achieve food security, stating that “a world where every person has economic and physical access to sufficient food to sustain a healthy and productive life, where malnutrition is absent, and where food originates from efficient, effective, and low-cost food and agricultural systems that are compatible with sustainable use and management of natural resources” [12] (p357). Many policies, priorities, technologies, and long-term solutions must be developed and implemented worldwide to achieve the 2020 food security vision [10,11,12]. However, there is a scarcity of systematic studies analyzing the food security drivers and the recommended policies to improve food security.

Following a review of the academic literature, we discovered a scarcity of research that systemically summarizes the major drivers of food security, outlines the recommended policies to improve food security, ensures the sustainability of future food production, and provides policy recommendations to enhance food security based on a country’s context. In response to this gap in the literature, we carried out a comprehensive, reliable, systematic, and rigorous review of previous research from the last ten years in order to identify the most frequently mentioned drivers/policies in the scanned literature. The rationale behind this study is to identify and list food security drivers and the current state of recommended policies that address chronic food insecurity to ensure the sustainability of future food production, utilizing a systematic literature review (SLR) methodology. Moreover, we hope to identify drivers/policies in order to aid policy-makers in selecting the most appropriate policies based on each nation’s context (e.g., agricultural production, natural resource availability, climate, political stability, and so on). Most importantly, policy-makers can use the identified drivers of food security and the recommended policies in the literature to customize appropriate policies that ensure the sustainability of future food production and, hence, ensure food sustainability for future generations. Based on the evidence reported in the literature, the identified food security drivers and recommended policies will aid the policy- and decision-makers of various countries in sustainably improving the food security situation. The need to identify the main drivers of food security arises from the notable increase in households and individuals suffering from food shortages and insecurity globally [25]. Finally, the findings of this research will be used to inform the GFSI developers in order to include more comprehensive indicators expected to contribute to the sustainability of future food production.

## 2. Materials and Methods

This research aims to report on food security drivers and the current state of recommended policies that address chronic food insecurity in order to ensure the sustainability of future food production through the use of a systematic literature review (SLR) methodology. We highlight existing food security drivers and outline recommended policies to alleviate food insecurity following the Preferred Reporting Items for Systematic Reviews and Meta-Analyses (PRISMA) data screening and selection guidelines [26]. The extraction process was meticulously documented in order to ensure the transparency and replicability of this systematic literature review [27]. A panel of researchers was formed, following the systematic review guidelines [26], to define the research field and questions, select keywords and the intended databases, and develop the sets of inclusion and exclusion criteria.

The research began by formulating the research questions to guide this systematic review based on identified gaps in the literature, guiding us in an attempt to answer the following research questions:

Q1. What are the main drivers of food security?
Q2. What are the main recommended policies to alleviate food insecurity?

By answering these questions, this paper provides a reference that policy-makers and practitioners can use to identify the main drivers of food security and the recommended policies in the literature in order to customize and choose appropriate policies that ensure the sustainability of future food production. The identified food security drivers and recommended policies are expected to aid policy- and decision-makers in improving the state of FS. This study also provides a roadmap for future research based on the evidence reported in the literature.

A specific research criterion was used to ensure that the research sources selected were sufficient and comprehensive enough to capture all of the significant and salient points to adequately answer the research questions [26]. To this end, we provide a critical review of the existing literature that has been published in two databases—Scopus and Web of Science (WOS)—between 2010 and 15 March 2021, to answer the abovementioned research questions. The time limit was set to cover the period following the global financial crisis of 2008/2009 and its effect on rising food prices, increased unemployment rates, and increasing food insecurity worldwide [28,29,30]. This period allows for consideration of policies designed to ensure global food security following the food shortage crisis. The use of Scopus and Web of Science (WOS) databases helped us to include most potential published works in a broad scope of journals, thereby limiting the risks of bias and possible exclusions associated with the use of fewer journals.

We employed a set of identified keywords, which are summarized in detail in Table 1. A critical analysis was conducted regarding the most relevant concepts that are available in the literature and which affect each of the four dimensions of FS: Food availability, food access, food utilization, and food stability. For instance, the research string “Agrifood supply chain” OR “Agri food supply chain” OR “Agri-food supply chain” was added as a secondary search string, because food availability is highly dependent on the food supply chain and how well its activities are managed. The food supply chain is exposed to many factors that can negatively impact the country’s food security level, such as severe weather conditions [31,32]. Therefore, it is critical to consider some characteristics of the food supply chain, such as biophysical and organoleptic features, shelf life, transport conditions, production time, and storage, to efficiently and effectively manage it [33]. Effective supply chain management is seen as a significant contributor to gaining and enhancing industrial competitive advantage and efficiency at the company level, possibly impacting food security positively [34]. “MENA Region” OR “Middle East and North Africa” OR “Middle East” OR “North Africa” research string was added due to the severity of food insecurity there and to ensure the inclusion of papers that address the problem in these countries and propose strategies to overcome food insecurity. According to the GFSI data [25], MENA region countries are experiencing a decline in food security; moreover, the number of households and individuals suffering from food shortages and insecurity is dramatically increasing.

The research string “Sustainable supply chain” OR “Resilient supply chain” was added due to much research that stressed the impact of designing a proper supply chain structure due to its significant impact on the future improvement of its performance [33]. The central mission of the twenty-first century is to establish a sustainable food system, which calls for a more concrete policy framework than what is currently in place [17]. Sustainability can be viewed as a prerequisite for long-term food security [1]. The environment, particularly climate and the availability of natural resources, is a prerequisite for food availability and biodiversity protection [24]. The availability of food for everybody depends on economic and social sustainability. Food utilization, too, is influenced by social sustainability. The three components of sustainability—social, economic, and environmental—assure the continuity of the three food security dimensions and the food system stability on which they rely. Moreover, food security is increasingly considered a prerequisite for long-term sustainability [1]. Adopting a “sustainable production and consumption approach throughout the global food supply chain” is a solution that will help reduce the amount of food waste along the food supply chain [35,36]. Cooper and Ellram [37] argued that building a resilient supply chain has many advantages such as decreasing inventory time, which will lead to cost and time savings, increasing the availability of goods, reducing the order cycle time, improving customer service and satisfaction, and gaining a competitive advantage. Stone and Rahimifard [38] stressed the importance of having a resilient agricultural food supply chain to achieve food security due to the incremental increase in volatility across the supply chain.

The research string “Food Safety” OR “Food diversity” OR “Food quality” OR “Food standards” OR “Micronutrient availability” was added due to one of the food security dimensions: utilization, which is concerned with all aspects of food safety, and nutrition quality [39]. According to FAO (2019), the utilization dimension should assess food diversity, food safety, food standards, and micronutrient availability. It is inadequate to provide enough food to someone unable to benefit from it because they are constantly sick due to a lack of sanitary conditions. It indicates that in the country, individuals are taking advantage of the food they receive or have access to, with extra emphasis on the dietary quality that contains nutritious ingredients such as vitamins (vitamin-A) and minerals (Iron, Zinc, Iodine) [40]. According to the World Health Organization, people diagnosed with malnutrition usually suffer from micronutrient deficiencies, protein deficiency, obesity, or undernutrition. The lack of micro-ingredients can increase the risk of developing severe chronic and infectious diseases for people in general and children in particular (toddlers 9–24 months). These diseases have an irreversible negative impact on people’s health, which enhances the persistence of poverty and food insecurity. It is critical to invest in the health and nutrition elements on a global scale by ensuring safe drinking water, immunization, enhancing sewage discharge, improving public health services, and reducing poverty levels [41].

The research string “Agricultural infrastructure” OR “Agricultural production volatility” OR “Vulnerability assessment” was chosen because much research has emphasized the importance of investing in a strong agricultural infrastructure to improve food security levels, especially in light of current challenges such as climate change, increased urbanization, water scarcity, and the shift away from using cropland for non-agricultural activities [7,8,41]. Food security is vulnerable to severe weather conditions, whereas harsh weather conditions may adversely impact the food supply chain in weak areas [31,32]. Therefore, it is critical to assess the vulnerability level of each country to protect the food supply chain. The use of the “Food loss” OR “Food waste” OR “Food waste and loss” research string was due to the general agreement among researchers on the importance of reducing food waste to improve food security [35,42,43]. According to the Food and Agriculture Organization (2013), around one-third of the food produced globally (1.3 billion tons) is wasted or lost. Most wasted food is either fresh and perishable or leftovers from eating and cooking [36,42]. Basher, Raboy [43] argued that eliminating just one-fourth of the food waste would be enough to feed all the currently undernourished people. One of the Sustainable Development Goals established by the United Nations, “SDG 12.3 Food Waste Index” stresses that decreasing the amount of food loss and waste will help reduce hunger levels, promote sustainable production and consumption, and enhance food security [44].

The use of “Policy description” OR “Policy assessment” OR “Policy recommendation” OR “Policymaking” OR “Policy-making” OR “Policy making” research string was due to the impact of adequate and proper policy formulation on food security (Table 1). Establishing effective and efficient food policies that ensure that each individual has an optimal level of food security is critical in every country because it directly enhances the country’s competitive advantage and efficiency [34,45]. Timmer [46] emphasized that designing the proper set of policies to end hunger based on each country’s context is challenging and requires collaborative participation from multiple stakeholders. Murti Mulyo Aji [34] stressed the role of the government’s policies in developing a collaborative supply chain that creates value throughout the supply chain by improving information, logistics, and relationship management. Effective and efficient supply chain management significantly impacts managing long-term partnerships and corporations among a wide range of firms that vary in size and sectors (public or private). This collaboration will enhance prediction of changes in customer demands in domestic and international markets. If previous policies were insufficient to ensure that country’s true competitive advantage, it could cause market distortion [34,47]. Countries are encouraged to gradually reduce the adoption of inequitable trade policies to focus on enhancing their true competitive advantage, demonstrating fair competition, and increasing economic efficiency, particularly in the spirit of trade liberalization [34].

The selection of research sources was accomplished in March 2021, and the search for keywords was enabled for titles, abstracts, and full texts in both electronic search engines (i.e., Scopus and WOS). Several keywords were identified to retrieve the available literature, and search strings consisted of primary and secondary keywords. The primary search string used was as follows: “food security” OR “food insecurity” OR “food availability” OR “food affordability” OR “food access” OR “food utilization” OR “food stability”. The reason behind including these multiple strings was to cover the maximum number of articles that handle the topic of food security or any of its four dimensions.

Specific exclusion and inclusion criteria were applied in order to develop high-quality evidence [26]. A reasonable number of articles were limited for deep analysis by following the specific exclusion and inclusion criteria to control the quality of the review in the food security field, as detailed in Table 2 above. Only peer-reviewed journal articles were included within the time frame (2010–15 March 2021) and only those written in English. Furthermore, due to this study’s nature and to ensure consistency with the topic area, the most common and effective approach for examining drivers and recommended policies were limited to the business, management, accounting, and agricultural fields [48]. We have used the “business, management and accounting” research field in the Scopus database to ensure that all the included articles were business-related. Then, we restricted the research field to” Economics, business, and agriculture Economics” in the WoS database to ensure the inclusion of agriculture-related papers and maximize the inclusion of a diverse range of articles. Another round of retrieval was applied using a set of secondary keywords in order to narrow down the search to specific areas of food security. For this purpose, the primary keywords were escorted each time with “AND” and other secondary keywords, as listed in Table 2.

**Figure 1:**
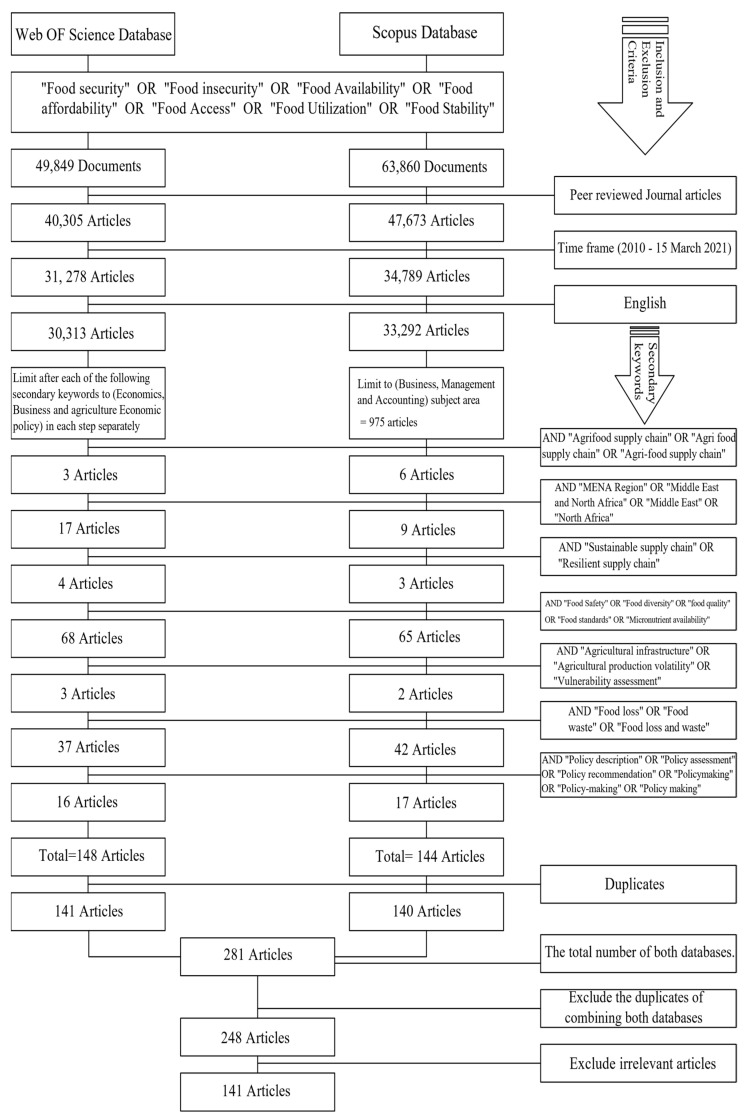
Research protocol following the PRISMA guidelines.

The initial search using the primary keywords (“food security” OR “food insecurity” OR “food availability” OR “food affordability” OR “food access” OR “food utilization” OR “food stability”) revealed a total of 113,709 documents (Scopus, *n* = 63,860; WOS, *n* = 49,849). Strict selection criteria were applied to the first search pool in order to maintain transparency and guarantee the selection of relevant material that answers the research questions. To ensure academic rigor, the search was restricted to including only peer-reviewed publications [49] (Scopus, *n* = 47,673; WOS, *n* = 40,305). The research was then restricted by publication date to between 2010 and 15 March 2021 (Scopus, *n* = 34,789; WOS, *n* = 31,278). Only journal articles published in English were selected (Scopus, *n* = 33,292; WOS, *n* = 30,313). Then, advanced research was conducted by combining the primary keywords with one of the secondary keywords. The results and the number of articles identified in each search step are detailed in Figure 1. After removing duplicate articles from each database, a total of 281 journal articles (Scopus, *n* = 140; WOS, *n* = 141) were revealed. After combining both databases, 248 journal articles were obtained. These collected 248 journal articles were scanned by reading their abstracts in order to check their applicability to answering the research questions. At this point, 107 articles were excluded as they were considered irrelevant and outside the scope of the research. Finally, the total number of extracted articles was 141, as can be seen in Figure 1. Data extraction and analysis were performed by a single reviewer (SW), and all extracted data and revealed results were double-checked by three researchers (FA, IM, and BS) to enhance the research and reduce bias in study selection. A complete description of the validity threats (Construct, Internal, External, and Conclusion Validity) following the validation process of Zhou, Jin [50] is provided in detail in Table 3.

**Figure 2:**
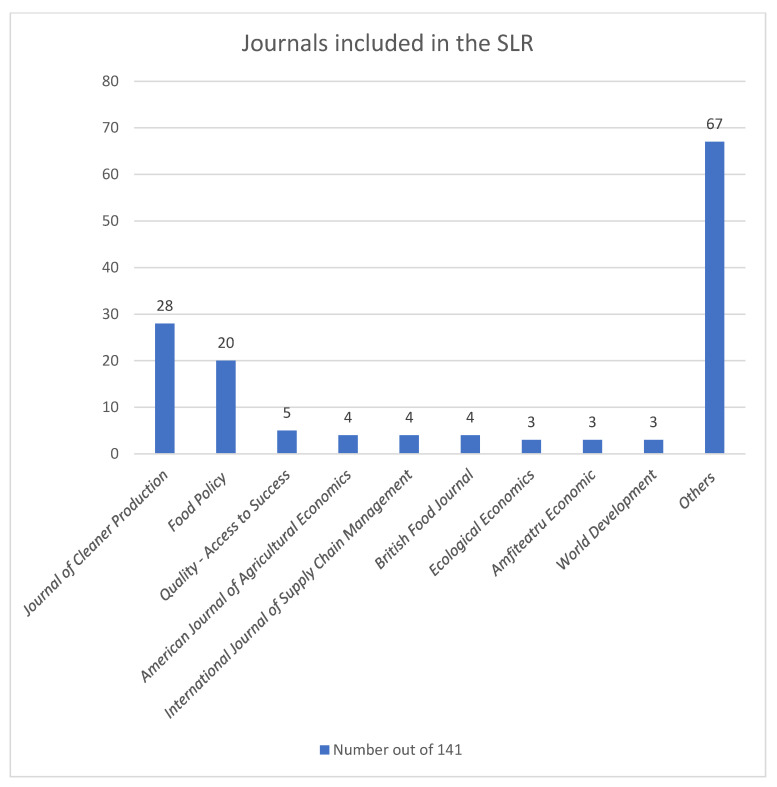
The most popular journals publishing the 141 included articles. Others denotes journals that were cited once or twice.

Among the selected 141 articles, 28 (19.86%) were published in the *Journal of Cleaner Production*, 20 (14.18%) were published in *Food Policy*, and 5 (3.55%) were published in *Quality-Access to Success*. The rest of the journal names are visualized in Figure 2.

## 3. Results

After the 141 articles have been extracted, they were analyzed and summarized individually by listing all the discussed food security drivers, as well as the recommended policies for the improvement of food security and sustainable food production. Then, we synthesized the extracted information from all sources in order to identify the gaps, list the similarities between all the resources, and extract significant insights regarding the main drivers of food security and the recommended policies [26].

### 3.1. The Major Drivers of Food Security

**Figure 3:**
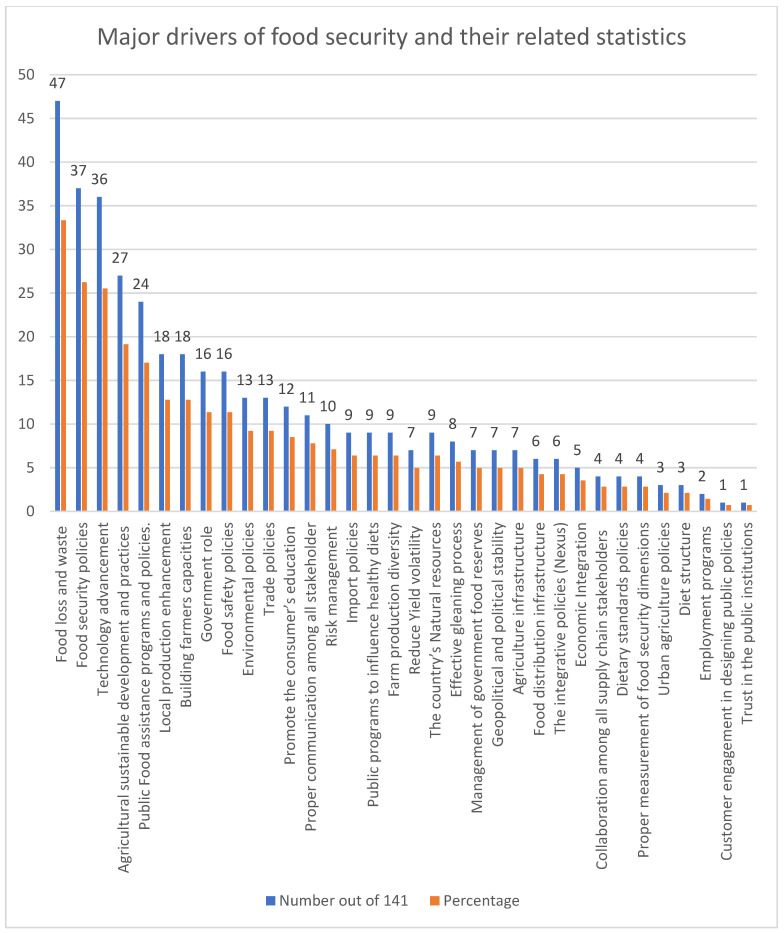
Summary of the major drivers of food security.

Analysis of the retrieved literature revealed 34 different drivers of food security, as visualized in Figure 3. Detailed information, along with a full citation list for all the drivers, is provided in Appendix A.

Most papers discussed food loss and waste (FLW) and emphasized its impact on food security [6,19,51,52,53,54,55,56,57,58,59,60,61,62,63,64,65,66,67,68,69,70,71,72,73,74,75,76,77,78,79,80,81,82,83,84,85,86,87,88,89,90,91,92,93,94,95]. Around one-third of the food produced globally (1.3 million tons) is wasted or lost [96]. Basher, Raboy [43] has argued that, if we could save just one-fourth of the wasted food, it would be enough to feed all the world’s undernourished people, contributing positively to FS. The previous finding supports our research findings that FLW is the primary driver of FS. To reduce FLW, Halloran, Clement [6] has argued that effective communication, more efficient food packaging, and a better consumer understanding of food packaging could lead to solutions. To decrease food loss, Garcia-Herrero, Hoehn [62] has suggested improving food labelling, enhancing consumer planning, and developing technological advances in packaging and shelf life for perishable products. Morone, Falcone [83] has suggested the repetition of large-scale research to help define a set of policies encouraging the transition to a new model for consumption that promotes sustainably procured food and dramatically reduces the amount of waste (more details are provided in Section 3.2).

Additionally, several authors have considered food security policy (FSP) as a driver of food security in its different forms [56,63,65,69,70,74,79,85,94,97,98,99,100,101,102,103,104,105,106,107,108,109,110,111,112,113,114,115,116,117,118,119,120,121,122,123,124]. The primary goal of establishing food security policies that consider the factors influencing individuals and groups is to reduce poverty and eliminate hunger. One example is safety-net programs or public food assistance programs (FAPs). The main goal of providing safety-net programs is to increase food consumption among poor people and improve food security [102].

Many papers have discussed the importance of technological advancement as an enabler of food security [56,57,58,63,69,71,74,77,85,90,94,95,109,116,119,120,121,123,124,125,126,127,128,129,130,131,132,133,134,135,136,137,138,139,140,141]. The use of technology to promote behavioral changes has increasingly become a vital instrument to reduce food waste and indirectly improve food security [130]. Mobile applications offer households helpful guidance on increasing shelf life and experimenting with dishes using leftovers [58]. Shukla, Singh [130] has elaborated that, at present, farmers have access to mobile applications that provide them with reasonably and timely priced information.

Some authors have discussed sustainable agricultural development and practices as enablers of food security [56,57,59,64,71,73,94,97,105,109,111,119,120,121,124,130,132,134,136,137,139,142,143,144,145,146,147]. Some authors have discussed local production enhancement as a driver of food security to enhance the self-reliance of countries [57,69,85,87,89,94,98,103,105,109,112,117,120,134,137,144,148,149]. For example, Ahmed, Begum [98] has emphasized how, following the GCC ban, Qatar took several successful steps to foster local production, support domestic businesses, and promote the consumption of locally produced food by its citizens. Some authors have argued that building the capacities of small farmers is essential to achieving FS. Education policies are critical for educating farmers, building their capacities, and increasing their human capital; moreover, educational programs should also include food preparation and health education programs in order to ensure the safety of consumed food [101].

The government’s role in managing a country’s agriculture can also be seen as a driver of food security [67,75,84,86,100,109,116,117,119,121,137,138,147,150,151,152], as it is responsible for various aspects such as designing, testing, and implementing the right policies to ensure the welfare of its citizens, while providing the necessary assistance to small-scale farmers and ensuring their safety and security in all aspects of life. Governments in developing nations must focus on R&D, agriculture infrastructure (e.g., technologies for irrigation and soil preservation), expansion services, early warning systems, or subsidized farm income in order to alter the production function of the population [101].

Many authors have discussed the importance of food safety policies as an enabler of food security [61,64,69,103,105,111,112,129,149,153,154,155,156,157,158,159]. Food safety policies include food and water safety at several points throughout the supply chain where food-borne diseases might develop [69]. Environmental policies are also seen as a fundamental enabler of food security [59,73,121,124,130,135,139,147,159,160,161,162,163]. Regardless of the various approaches discussed by the authors, they all agreed that environmental protection would help to ensure food availability for current and future generations. According to some authors, trade policies [69,94,95,103,111,112,114,123,129,141,146,161,164] and import policies [69,95,100,103,120,124,126,129,146] are enablers of food security. Regulating international trade can help to ensure food security. Lowering trade barriers, for example, has been proposed as a way to mitigate the adverse effects of market regulation caused by climate change [141].

Many authors have recognized policies that promote consumer education on sustainable consumption and increase consumer awareness and knowledge of the environmental impact of their purchases as a driver of food security [52,60,67,69,86,133,144,151,163,165,166,167]. Others have stressed proper communication among all stakeholders as a driver of food security [6,56,68,69,84,92,129,130,156,157,168]. Some authors have considered risk management as an enabler of food security [94,117,118,137,138,139,145,154,155,157]. For example, the aims of building a disaster risk reduction framework in the Pacific include boosting resilience, protecting investments (e.g., in infrastructure, operations, and FS), and decreasing poverty and hunger [169].

Some authors have proposed the effective gleaning process as a driver of food security [70,72,74,80,84,92,142,170]. Gleaning is the collection of the remaining crops in agricultural fields after their commercial harvest, or just in crop fields where their harvest is not cost-effective. Some old cultures have fostered gleaning as an early form of social assistance [80]. Some authors have considered the management of government food reserves to be a food security driver [64,104,112,117,118,124,136]. Despite the high cost of storing food, any country must maintain adequate food reserves to serve the country in case of a crisis scenario [171]. Some authors have considered integrative policies (i.e., food–water–energy, food–energy, or water–food) as a driver of food security due to their impact on environmental improvement through natural resource handling efficiency [56,73,133,139,172,173]. Some authors have considered establishing dietary standard policies as an enabler of food security [69,151,163,174]. The government should impose policies on healthy food consumption to prevent obesity, such as prohibiting trans-fats. Moreover, they should restrict trans-fat usage in food outlets, establish institutional food standards, implement menu labelling regulations for chain restaurants, and ensure that disadvantaged people have better access to healthy meals [151].

Authors have highlighted various additional arguments or policies that are considered drivers for FS such as establishing public programs to influence diets in a healthy manner, reducing yield volatility [85,94,105,119,124,126,175], the country’s natural resources [85,105,119,124,137,145,162,163,176], geopolitical and political stability [69,98,104,117,123,124,142], agricultural infrastructure [64,114,116,118,142,146,175], food distribution infrastructure [71,75,76,112,177,178], economic integration [109,112,123,179,180], collaboration among all supply chain stakeholders [75,130,134,157], proper measurement of food security dimensions [123,181,182,183], urban agriculture policies [56,147,148], adjustments in dietary structure [59,86,163], establishing employment programs for poor household representatives [110,152], customer engagement in designing public policies [158], and trust in public institutions [166].

### 3.2. The Recommended Policies to Alleviate the Food Insecurity

**Figure 4:**
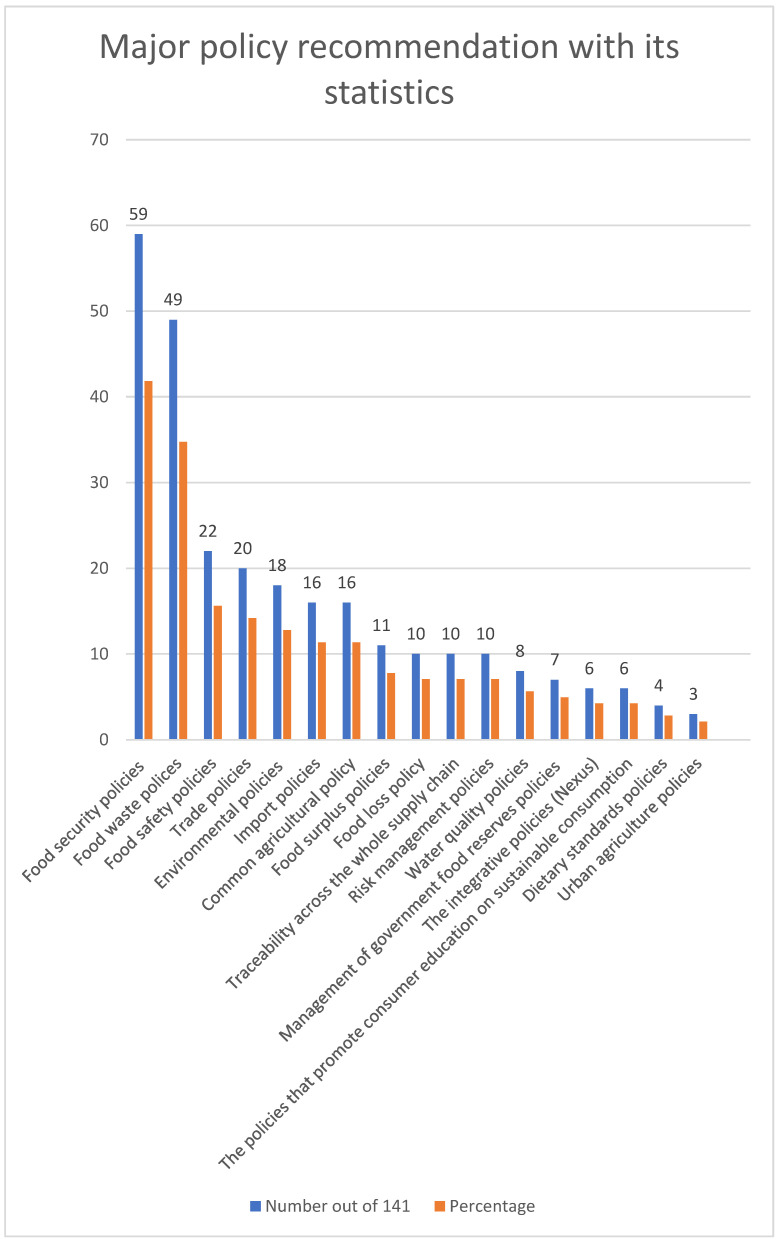
The main 17 recommended policies and statistics.

Analysis of the 141 retrieved papers revealed 17 major recommended policies, as visualized in Figure 4. We also determined sub-policies under each category which were grouped based on common characteristics, relevance, and how they were categorized in the papers. The complete list of sub-policy categories and related references is provided in Appendix B.

Most authors recommended establishing FSP, in general, as a primary solution for food insecurity in developing and developed countries [56,57,63,64,65,69,81,85,87,89,91,94,97,98,99,101,102,103,104,105,106,107,108,109,110,111,112,113,114,115,116,117,118,119,120,121,122,123,124,126,127,130,131,133,134,137,142,144,145,148,149,151,152,175,177,180,182,184,185]. Many authors have suggested food consumption policies that offer safety-net programs or public food assistance programs (FAPs) such as food price subsidies, cash-based programs, structural pricing adjustments, or micro-credits as enablers of FS. The main goal of providing safety-net programs is to increase food consumption among poor people and improve food security [102]. Given the solid bidirectional causal link between poverty and malnutrition, FAPs have been recognized as critical components of the overall poverty reduction strategy. Food aid policies and initiatives can fill the gaps left by the for-profit food system and the informal (non-profit) social safety nets, ensuring food security for disadvantaged individuals, families, and communities [108]. Several authors have recommended establishing policies to enhance the performance and asset bases of small-scale farmers, such as loans, subsidies, access to information, and knowledge-sharing, to address food insecurity. Governments should adopt direct interventions such as structural price adjustments and targeted food subsidies to enhance the food access of farmers by lowering market prices and stabilizing consumption during high food price inflation [116]. Others have recommended establishing government input subsidy programs (input subsidy policies) that provide farmers with subsidies for investment into high-yielding technology (e.g., automation, fertilizers, high-yield seed). They all claimed this as an effective policy instrument for agricultural development, but each focused on a different mechanism. Shukla, Singh [130], for example, has discussed public distribution programs; Sinyolo [131] has emphasized policies aimed at increasing the amount of land planted with enhanced maize varieties among smallholder farmers; Wiebelt, Breisinger [124] has suggested investments in water-saving technologies, while Tokhayeva, Almukhambetova [137] have proposed the development of an agricultural innovation system. Others have recommended rural development policies to reduce yield volatility and improve the agricultural infrastructure (e.g., irrigation and water-saving technologies). Governments in developing nations must focus on R&D, agricultural infrastructure (technologies for irrigation and soil preservation), expansion services, and early warning systems [101]. Technological advancement, in general, is seen as a vital element in reducing yield volatility [85]. Capacity-building policies (e.g., educational, training, and technical support) have received considerable attention in the literature as a fundamental component of urban farming initiatives, and as attempts to promote self-reliance and networking. Capacity building in many areas connected to urban agriculture is essential for equipping residents with knowledge and expertise [148]. To enhance FS, some researchers have suggested policies supporting locally produced food, diversified agricultural production policies, policies that impact farm-level commodity pricing, food stock policies, establishing policies to increase the income of farmers, buffer stock policies, and resource allocation policies (for a complete list of references, see Appendix B).

Many authors have proposed different policy recommendations to reduce food waste and, thus, food insecurity [6,19,51,52,56,57,58,60,61,62,63,64,65,66,67,68,69,70,71,72,73,74,75,76,77,79,80,81,82,83,84,85,86,87,88,91,92,93,94,103,130,138,144,150,160,167,168,170,177]. Many have agreed on the importance of policies that promote information and education campaigns that spread awareness at household and public levels by improving meal planning and management in consumers. However, each author suggested a different approach. For example, Schanes, Dobernig [58] have discussed face-to-face door-stepping campaigns (online and in traditional newspaper leaflets), word-of-mouth, and television shows or movies. However, Septianto, Kemper [66] have highlighted the importance of social marketing campaign design and framing (having vs. not having) in conveying the intended message to consumers. Tucho and Okoth [73] have asserted the advantages of producing bio-wastes and bio-fertilizers from food waste and human excreta (in a food–energy–sanitation nexus approach), and also advocated for educating families on how to do so at the household level. Xu, Zhang [86] has argued that governments should help society to develop a logical perspective on food consumption and aggressively promote the habit of eating simple meals, particularly in social catering. Von Kameke and Fischer [52] and Zorpas, Lasaridi [60] have emphasized the importance of teaching customers about efficient meal planning to reduce food waste. Von Kameke and Fischer [52] have proposed using the Nudging tool rather than campaigning. Xu, Zhang [86] have suggested initiating suitable policy instruments to nudge individuals to adopt sustainable consumption habits, with important implications for decreasing food waste and increasing food security in China. Smart (innovative) food packaging and labelling policies have received significant attention in the literature, as they are critical in reducing food waste and, thus, improving FS. The nature, size, and labelling of the packaging impact the lifetime of the food. Smart packaging innovations and new technologies are steadily penetrating markets, thus increasing the shelf-life of foods through enhanced protection, communication, convenience, and control [58].

Food banks, food sharing, and food rescue policies have also received significant attention in the global literature, as they help reduce food waste and improve FS. Food banking is a critical long-term rescue policy for re-distributing surplus food to those in need and reducing poverty and food insecurity [80,92]. Several authors have recommended positive sanctions such as financial rewards, tax credits, federal and state funding, vouchers, or reduced taxes to decrease food waste and improve FS. Positive sanctions consist mainly of financial incentives to encourage restaurants and grocery retailers to donate their leftover food [60]. Addressing liability concerns might be one incentive, as the research participants have highlighted this as a universal barrier and that this issue, in particular, must be handled [51]. Negative sanction policies have received considerable attention in the literature as a tool for reducing food waste and improving FS. These include fines and fees imposed on companies and individuals accountable for food waste [58]. Taxes and fines are a potential way to manage and motivate restaurants and retailers to donate their leftover food to charities and community centers [65].

The establishment of policies that regulate the sharing of information and knowledge among supply chain stakeholders has received some attention in the literature in terms of reducing food waste and improving food security. Comprehensive food waste legislation has been discussed as a potential enabler of food security. A possible regulatory tool would be to revise and remove unnecessary food safety requirements that result in excessive food waste levels [58]. According to Halloran, Clement [6], food waste increased due to European food safety regulations and standardization. Food waste recycling policies have been used as a method to reduce food waste. Food waste can be utilized for value generation at any point of the food supply chain process through efficient techniques, then reincorporated into the cycle [77]. Food waste has a long history as a source of ecologically friendly animal feed [61].

A few authors have highlighted the impact of technological advancement (e.g., mobile applications) as a strategy to reduce food waste. Some authors have proposed implementing gleaning operation policies that provide tax incentives and government assistance to gleaners in order to decrease food waste. Some authors have proposed implementing peak storage reduction policies, such as stock-holding incentives. Nudging tools (which nudge people toward forming sustainable consumption behaviors) have been mentioned by a few authors.

Food safety policies received significant attention in the retrieved literature [61,64,69,70,103,105,111,112,120,125,129,130,137,138,149,153,154,155,156,157,158,159]; however, they have been discussed in various different forms. Few authors have discussed food quality and food hygiene compliance certifications. Compliance with sanitary standards is required to maintain the best practices for preventing food-borne diseases and food security threats [155]. Other authors have discussed the importance of food safety standards. Meanwhile, few authors have emphasized the importance of food safety throughout the supply chain, but each proposed a different strategy to achieve it. For example, some authors have suggested using an effective IT system [130], RFID [138], or developing food safety training policies [155].

Many authors have advocated for the implementation of trade policies to address food insecurity in developing and developed countries [94,95,101,103,111,112,119,123,129,136,141,146,148,149,152,157,161,164,178,180], but in different contexts. For example, some have suggested establishing infrastructure development policies that target agricultural logistic infrastructure, or improving the speed and quality of shipping logistics. In contrast, some authors have agreed on the importance of state trading and private trade-supporting policies. Others have suggested the removal of tariff and non-tariff barriers, while a few authors recommended reliable marine connection and transportation logistics policies.

Environmental policies are a fundamental enabler of food security [59,73,94,120,121,124,130,135,139,141,145,147,159,160,161,162,163,166]. However, authors have focused on many different aspects of these policies. Some authors, for example, have emphasized the importance of establishing policies to mitigate the effects of climate change. Others were too specific, suggesting greenhouse gas reduction policies, and proposed penalizing non-compliance. Due to the strong links between climate change, poverty, and food insecurity, some authors have proposed establishing coordinating policies among the three. Other authors have stressed the consideration of policies that encourage the optimization of fertilizer use.

Many authors have considered food import policies as a solution to food insecurity [94,95,100,103,104,105,109,112,116,117,119,120,124,126,134,146]; however, most authors provided different opinions regarding the most effective policy to implement. For example, some authors have stressed the importance of policies that provide direct government financial assistance to local agriculture, or the importance of policies that sustain local agricultural product prices compared to imported products. Some have recommended providing temporary tax benefits for agricultural investment, while others recommended import ban (substitution) policies. A few authors have recommended direct budget subsidies, subsidized loan interest rates, and strategies for the diversification of imported food origin.

Many authors have discussed the importance of establishing a common agricultural policy (CAP) to address sustainable agriculture [56,57,64,89,109,111,118,119,132,142,143,149,161,172,184,186]. Others have stressed the importance of food surplus policies in enhancing a country’s food security status [51,58,70,72,75,76,79,82,84,90,91]. Some authors have suggested strategies to regulate a company’s liability regarding the donation of surplus food. A few authors have proposed food policies that subsidize the purchase of surplus food—also known as “ugly food”—by controlling for prices and surplus item characteristics. Some authors have suggested establishing food loss policies. However, few authors have specified the need for policies promoting food loss quantification.

Many authors have discussed the policies that promote traceability across the whole supply chain as an enabler for food security [56,69,103,128,129,130,137,138,168,178]. However, the different authors discussed different technologies such as investment into information technology such as RFID, effective IT systems, ICT systems, and blockchain technology. Government policies should promote investments into traceability systems that focus on rapid withdrawal in unsafe food scenarios such as product recall regulations, fines imposed on hazardous product distributors, and food-borne food risk monitoring [129]. Many authors have discussed various risk management strategies to improve a country’s food security [94,117,118,137,138,139,145,154,155,157]. However, each considered a different approach to overcome the risk. Specifically, they have discussed food scandal policies, the COVID-19 pandemic, programmed risk identification, proactive policy measures to handle flood crises, early warning systems for natural disasters, or risk management throughout the food supply chain. Some authors have highlighted water quality policies such as efficient water-use policies, improving water resources policies, using water-efficient crops, investments into water-saving technologies, and food and water safety throughout the supply chain.

Some authors have discussed the management of government food reserves as an enabler of food security [64,104,112,117,118,124,136], and others have discussed integrative and coherent policies between food, water, and energy (as a nexus) [56,73,133,139,172,173]. Meanwhile, other authors have discussed policies that promote consumer education on sustainable consumption, improving consumer status awareness and knowledge regarding the ecological impact of their purchases [60,69,133,144,163,165]. Few authors have addressed the importance of dietary standard policies [69,151,163,174], urban agriculture policies [56,147,148], and food-aid policies [118,150].

Some policies were suggested in one paper only such as devising the right population policy in China [85], flexible retail modernization policies [158], policies that facilitate short-term migration [187], policies to stimulate equitable economic growth through manufacturing and services [95], and sound research governance policies [140].

## 4. Discussion

In this section, we discuss the polices and drivers in the greater areas, then compare them based on specific contexts. This approach serves to provide better understanding, thus informing decision-makers about the importance of choosing the right policies through considering many food security dimensions. By looking deeply at the extracted food security drivers and policies and the way in which they can be applied to each country’s context, we take an example from the MENA region. The MENA region includes a diverse range of nations, including low-income and less-developed (e.g., Sudan, Syria, and Yemen), low–middle-income (e.g., Algeria, Egypt, Iran, Morocco, and Tunisia), upper middle-income (e.g., Jordan, Lebanon, and Libya), and high-income (e.g., the UAE, Qatar, Oman, Bahrain, Israel, Kuwait, and Saudi Arabia) countries [126]. As food availability is a serious problem in the MENA region low-income countries (Syria and Yemen), due to war and violent conflicts [188], policies aimed at increasing food availability continue to pique the interest of policy-makers. In these countries, where citizens are incapable of fulfilling their basic food needs [189], the existence of food security policies in different forms is crucial for achieving food security [53,97,98,124,184], more than FLW policies. Policy-makers should focus on ensuring the availability of either locally produced or imported food, which requires appropriate trade policies to deal with food shortages and improve the availability dimension in these countries. Trade policies should focus on creating infrastructure development policies that target agricultural logistic infrastructure, improve the speed and quality of shipping logistics, and establish reliable marine connections and transportation logistics policies that remove tariff and non-tariff barriers.

Policy-makers should establish import policies that sustain local agricultural product prices compared to imported products, provide direct government financial assistance to local agriculture, and provide temporary tax benefits for agricultural investment.

Additionally, the governments should improve food access in the MENA region low-income countries by reducing or stabilizing consumer and producer food prices. To enhance food access, FSPs (e.g., education policies in general and capacity-building policies) may help to improve individual human capital. Governments also must provide supplemental feeding programs, typically targeting vulnerable groups in need of special diets, such as pregnant women and children [101].

Moreover, the government should improve credit access through the following means: policies that enhance the performance and asset base of small-scale farmers; the existence of policies that impact farm-level commodity pricing, thus retaining farmers and increasing local production; the existence of government input subsidy programs for individuals, and the existence of policies supporting locally produced food. These are all possible policies to improve the MENA region FS. Governments and global health organizations should promote food utilization in MENA low-income countries through the development of policies that monitor overall food quality, such as access to clean water and micronutrient fortification, or through individual educational programs on safe food preparation [155]. Finally, enhancing food quality can optimize the individual nutrient absorption [101].

In contrast, discussions of food security in the MENA region high-income countries have indicated that food availability, access, and utilization are generally higher and not a problem. However, food stability is low, which requires the attention of policy-makers to improve FS. Food stability impacts the other food security pillars (access, availability, and utilization). Moreover, it requires the economic, political, and social sustainability of food systems, which are vulnerable to environmental conditions, land distribution, available resources, conflicts, and political situations [190]. Food stability necessitates increased efforts and expenditures to achieve food security in the sustainable development goals, especially in light of increased academic and governmental interest in incorporating sustainability values into policies.

As food waste is prevalent in these countries, FLW policies are more critical than FSP, which is in alignment with our findings regarding food security drivers. FLW makes it difficult for the poor in developing countries to access food by significantly depleting natural resources such as land, water, and fossil fuels while raising the greenhouse gas emissions related to food production [115]. Addressing food loss and waste in these countries can hugely influence the reduction of wasted food and indirectly enhance food security. The number of food-insecure individuals may be reduced in developing regions by up to 63 million by reducing food loss, which will directly reduce the over-consumption of cultivated areas, water, and greenhouse gas emissions related to food production [115]. According to Abiad and Meho [189], food waste produced at the household level differs across MENA-region countries. For example, it ranges from 68 to 150 kg/individual/year in Oman, 62–76 kg/individual/year in Iraq, 194–230 kg/individual/year in Palestine, and 177–400 kg/individual/year in the UAE. It is critical to take more aggressive but scientifically sound initiatives to minimize FLW, which will require the participation of everyone involved in the food supply chain such as policy-makers, food producers and suppliers, and the final consumers [191,192]. Food waste reflects an inefficient usage of valuable agricultural input resources and contributes to unnecessary environmental depletion [191,193]. Furthermore, food loss is widely recognized as a major obstacle to environmental sustainability and food security in developing nations [194]. Preventing FLW can result in a much more environmentally sustainable agricultural production and consumption process by increasing the efficiency and productivity of resources, especially water, cropland, and nutrients [115,191,192,195]. Preventing FLW is crucial in areas where water scarcity is a prevalent concern, as irrigated agriculture makes up a sizeable portion of total food production, and yield potential may not be fully achieved under nutrient or water shortages [191,196,197]. According to the study of Chen, Chaudhary [197], food waste per capita in high-income countries is enough to feed one individual a healthy balanced diet for 18 days. Chen, Chaudhary [197] also found that high-income countries have embedded environmental effects that are ten times greater than those of low-income countries, and they tend to waste six times more food by weight than low-income countries. Consequently, implementing proper FLW policies in high-income countries can help to alleviate the food insecurity problem while maintaining the economic, social, and environmental sustainability of future food production.

Implementing effective food storage techniques and capacities is considered a key component of a comprehensive national food security plan to promote both food utilization and food stability; furthermore, proper food storage at the household level maintains food products for a more prolonged period [198]. Encouragement of economic integration between MENA region countries is very applicable considering the heterogeneity of these countries. For example, countries with limited arable land and high income, such as the UAE and Saudi Arabia, can invest in countries with a lower middle income, such as Egypt, and use its land to benefit both countries. On the other hand, Boratynska and Huseynov [101] have proposed food technology innovation as a sustainable driver of food security and a promising solution to the problem of food insecurity in developing countries. Due to the higher food production demand to support the expanding urban population while having limited water and land availability, higher investments in technology and innovation are needed to ensure that food systems are more resilient [190]. Boratynska and Huseynov [101] have argued that, in general, using innovative technologies to produce healthy food products is frequently a concern. However, improving the probability that innovative food technology will enable the production of a diverse range of food products with enhanced texture and flavor while also providing a variety of health advantages to the final consumer is essential. Jalava, Guillaume [193] have argued that, along with reducing FLW, shifting people’s diets from animal- to plant-based foods can help to slow environmental degradation.

The MENA region example described above can be adapted to different regions based on their food security situation, and relevant policies can be devised to improve food security more sustainably.

## 5. Conclusions

Food security is a complicated and multi-faceted issue that cannot be restricted to a single variable, necessitating the deeper integration of many disciplinary viewpoints. It is essential to admit the complexity of designing the right policy to improve food security that matches each country’s context [46] while considering the three pillars of sustainability. Furthermore, it is of utmost importance to implement climate-friendly agricultural production methods to combat food insecurity and climate change [12]. Mapping the determinants of food security contributes to better understanding of the issue and aids in developing appropriate food security policies to enhance environmental, social, and economic sustainability.

This research contributes to the body of knowledge by summarizing the main recommended policies and drivers of food security detailed in 141 research articles, following a systematic literature review methodology. We identified 34 food security drivers and outlined 17 recommended policies to improve food security and contribute to sustainable food production. Regarding the drivers, one of the foremost priorities to drive food security is reducing FLW globally, followed by food security policies, technological advancement, sustainable agricultural development, and so on (see Appendix A). Regarding the recommended policies, most studies have detailed the contents and impacts of food security policies, food waste policies, food safety policies, trade policies, environmental policies, import policies, the Common Agricultural Policy (CAP), food surplus policies, and so on (see Appendix B).

### 5.1. Policy Implications

We assessed the obtained results in comparison to the latest version of the GFSI. Using the GFSI (2021) indicators as a proxy resulted in the identification of gaps and specific policy implications of the results. The idea was to identify which of the policies and drivers have been already implemented and which have not (or, at least, have not been very successfully implemented). We used the GFSI as it is a very well-established benchmarking tool used globally by 113 countries to measure the food security level. We examined the indicators mentioned under each of the four dimensions of food security, and listed associations with the identified policies and drivers found in the literature. Accordingly, we suggest the addition of two dimensions to the current index:

- Sustainability

The first dimension relates to measuring the sustainability dimensions that each participating country adopts in its food production process. We noticed that many authors stressed the importance of the existence of clear environmental policies that drive long-term food security. However, the current GFSI lacks indicators measuring this dimension. The reviewed literature suggested environmental indicators considering optimized fertilizer use, carbon taxes, aquaculture environment, bio-energy, green and blue infrastructure, gas emissions reduction policies, policies to reduce the impacts of climate change, and heavy metal soil contamination monitoring.

- Consumer representation

The second dimension is related to consumer voice representation within the GFSI. The reviewed literature suggested implementing policy measures that promote consumer education on sustainable consumption and improve the consumer status, consciousness, and knowledge regarding the ecological impact of their purchases. Any sustainability initiative should be supported and implemented by the final consumer.

Additional gaps in the policies and drivers of food security were identified and allocated under the relevant indicators in the GFSI based on the four dimensions of food security. Under the affordability dimension, we found a lack of policies in the reviewed literature addressing the Inequality-adjusted income index. Regarding the Change in average food costs indicator, we observed that the policies that exist in the literature concern the farmer level only (e.g., policies that impact farm-level commodity pricing and policies supporting locally produced food), and not all of the citizens at the national level. Additionally, policies that promote traceability across the whole supply chain were missing. There were no policies in the reviewed literature under the food quality and safety dimension representing the following: the dietary diversity indicator; micronutrient availability (e.g., dietary availability of vitamin A, iron, and zinc); regulation of the protein quality indicator; the food safety indicator (specifically the two sub-indicators of food safety mechanisms and access to drinking water), and illustration of the national nutrition plan or strategy indicator. Therefore, future research should pay more attention to and emphasize the importance of such policies, particularly in developed countries seeking to improve their food security status and score high on the GFSI.

Moreover, the reviewed literature suggested “developing food safety training policies” to improve food safety and FS; however, no indicators or sub-indicators within the GFSI represent such training policies. The GFSI developers should pay more attention to safety training practices and include them in the index’s future development. Under the availability dimension, the reviewed literature suggested establishing a food loss policy that promotes the quantification of food loss under the food loss indicator. This indicator should be enhanced through well-articulated policies that address the problem of food loss and attempt to mitigate its impact. However, while there were various policies concerning food waste or surplus, there were no indicators within the GFSI that represented food loss. As food loss and waste was identified as the primary driver of food security in this study, we recommend expanding the GFSI to include food loss quantification and reduction policies under the availability dimension. Finally, under the political commitment to adaptation dimension, some policies were identified in the reviewed literature in two sub-indicators: early warning measures/climate-smart agriculture (e.g., proactive policy measures to handle flood crises, programmed risk identification, and early warning systems for natural disasters) and disaster risk management (e.g., food scandals, COVID-19, and risk management throughout the food supply chain). However, under the other two relevant sub-indicators—commitment to managing exposure and national agricultural adaptation policy—there were no identified policies.

### 5.2. Contributions of the Study

The key contributions of this study to the existing literature are threefold. First, we identified the (34) main food security drivers and the (17) most-recommended policies to improve food security and enhance the future food production sustainability. Several studies have partially covered this area, but none have employed a systematic literature review of 141 papers covering such an scope in this topic. The gravity of food security worldwide is well established; hence the contribution of this work. Second, we provide a reflection of policies/drivers on the latest version of the GFSI, resulting in more tangible policy implications (see Section 5.1). Third, through a systematic literature review, we identified elements not listed under the GFSI that could be considered in its future revision. Examples include environmental policies/indicators such as optimized fertilizer use, carbon taxes, aquaculture environment, bio-energy, green and blue infrastructure, gas emission reduction, policies to reduce the impact of climate change, and heavy metal soil contamination monitoring; consumer representation, as the reviewed literature suggested policy measures that promote consumer education on sustainable consumption, as well as improving consumer status, consciousness, and knowledge regarding the ecological impact of their purchases; and traceability throughout the entire supply chain.

### 5.3. Study Limitations and Future Research

In this study, we identified the major drivers and the recommended policies to improve food security and enhance the future food production sustainability based on the reviewed literature. However, we recommend conducting a Delphi research study in consultation with policy-makers and industry experts. A Delphi study can be used to validate the findings of this systematic literature review based on a specific country’s context. This research was conducted using only 141 articles from two databases; therefore, we suggest replicating this research using different databases, which will allow for the inclusion of more related papers. Moreover, this research included only peer-reviewed articles, which may be considered, based on the guidelines of Keele [185], as a source of publication bias. Future research may consider including gray literature and conference proceedings. This research did not include the three sustainability pillars within its research string; therefore, we recommend considering the inclusion of the three pillars in future research. Future research should also investigate the use of alternative protein food technology innovation, such as plant-based protein, cultured meat, and insect-based protein, as a sustainable solution to the food security problem. Additionally, understanding the factors influencing acceptance of various technologies by the final consumer is particularly important given some regional characteristics such as harsh arid environments and the scarcity of arable land, freshwater, and natural resources.

## Figures and Tables

**Table 1:** Primary and secondary search strings used in this research.

Keywords	Primary or Secondary
(“Food security” OR “Food insecurity” OR “Food Availability” OR “Food affordability” OR “Food Access” OR “Food Utilization” OR “Food Stability”)	Primary search string
“Agrifood supply chain” OR “Agri food supply chain” OR “Agri-food supply chain”	Secondary search string
“MENA Region” OR “Middle East and North Africa” OR “Middle East” OR “North Africa”	Secondary search string
“Sustainable supply chain” OR “Resilient supply chain”	Secondary search string
“Food Safety” OR “Food diversity” OR “food quality” OR “Food standards” OR “Micronutrient availability”	Secondary search string
“Agricultural infrastructure” OR “Agricultural production volatility” OR “Vulnerability assessment”	Secondary search string
“Food loss” OR “Food waste”	Secondary search string
“Policy description” OR “Policy assessment” OR “Policy recommendation” OR “Policy making”	Secondary search string

**Table 2:** Inclusion and exclusion criteria.

Criterion	Inclusion	Exclusion
Study type	Only peer-reviewed journals, both empirical and theoretical/conceptual studies AND industry reports.	Any non-peer-reviewed journals, conference articles, magazines, news.
Language	English written sources.	Any other language.
Research field	Limit to business, management, accounting, and agriculture.	Exclude other fields.
Date	Until 15 March 2021.	Before 2010.
Relevance	Include relevant studies related to food security and food technology domains.	Exclude irrelevant studies.

**Table 3:** A reporting of validity threats in this systematic literature review.

The Validity	Taken Precaution
Construct Validity	The SLR setting was specified, and sufficient information was given. We ensured that appropriate and complete search terms were used in the automatic search. Two databases were used to extract articles answering the research questions. The correctness of the search method was checked by multiple authors. A thorough search strategy was used in conjunction with a multi-step selection process to ensure that the inclusion and exclusion criteria were appropriate. To ensure appropriate research question formulation, the researchers (experts in the research area) held several internal discussion meetings.
Internal Validity	The SLR setting was specified, and sufficient information was given. We ensured that appropriate and complete search terms were used in the automatic search. The correctness of the search method was checked by multiple authors. We used two databases to extract articles and a set of modified search keywords to ensure the appropriate sample size of the retrieved articles. The articles retrieved from the two databases were checked twice to identify and eliminate duplicates. Data extraction and analysis were performed by a single reviewer (SW). All extracted data and revealed results were double-checked by three researchers (FA, IM, and BS) to enhance the research and reduce the bias in study selection and extraction, as well as reducing subjective interpretation. The researchers came from three different cultural backgrounds, which helped to minimize the cultural bias.
External Validity	Precautions were taken into consideration to enhance the reliability and validity of the research; however, the research findings still need to be validated by replicating this research using different data sets and validating the result through three rounds of Delphi research. Such validation will boost the study’s generalizability. The researchers contacted some authors to obtain articles that were not accessible online. The systematic literature review provides objective, accurate, and in-depth information, presented in the analysis section.
Conclusion Validity	The articles retrieved from the two databases were checked twice in order to identify and eliminate duplicates. Data extraction and analysis were performed by a single reviewer (SW). All extracted data and revealed results were double-checked by three researchers (FA, IM, and BS) to enhance the research, reduce the bias in study selection and extraction, and reduce subjective interpretation.

## Data Availability

Not applicable.

## Appendix A. Summary Table of Major Drivers of Food Security

**The Driver of Food Security**	**Occurrence**	**Article Citations**
Food loss and waste	47/141	[6,19,51,52,53,54,55,56,57,58,59,60,61,62,63,64,65,66,67,68,69,70,71,72,73,74,75,76,77,78,79,80,81,82,83,84,85,86,87,88,89,90,91,92,93,94,95]
Food waste management	29/47	[19,51,52,53,54,55,56,57,58,59,60,61,62,63,64,65,66,67,68,69,70,71,72,73,74,75,76,77,78,79,82,84,93,167,199]
Food waste policies	23/47	[6,51,52,58,60,62,63,64,65,66,68,69,71,75,77,79,80,81,82,83,84,85,86].
Food loss reduction policies	10/47	[62,65,68,77,79,84,89,93,94,95].
Food surplus policies	11/47	[58,70,72,75,76,79,82,84,90,91,92].
Food waste quantification	11/47	[54,58,63,68,71,78,82,84,93,167,199]
food loss quantification	5/47	[68,82,87,88,89]
Food security policies	37/141	[56,63,65,69,70,74,79,85,94,97,98,99,100,101,102,103,104,105,106,107,108,109,110,111,112,113,114,115,116,117,118,119,120,121,122,123,124]
Environmental policies	13/141	[59,73,121,124,130,135,139,147,159,160,161,162,163]
Public food assistance programs and policies	24/141	[65,81,99,101,102,104,105,107,108,109,110,112,113,114,115,116,118,119,122,137,144,148,151,152,182]
Risk management	10/141	[94,117,118,137,138,139,145,154,155,157]
Food scandals policies	2/10	[154,155]
Early warning systems for natural disasters	3/10	[94,137,145]
Risk management throughout the food supply chain	3/10	[138,139,157]
Proactive policy measures to handle the flood crises	2/10	[118,145]
Providing food aids (micronutrient supplementation) during disasters	1/10	[118]
COVID-19 pandemic	1/10	[117]
The programmed risk identification	1/10	[139]
Import policies	9/141	[69,95,100,103,120,124,126,129,146]
Trade policies	13/141	[69,94,95,103,111,112,114,123,129,141,146,161,164]
Economic integration	5/141	[109,112,123,179,180]
Agricultural sustainable development and practices	27/141	[56,57,59,64,71,73,94,97,105,109,111,119,120,121,124,130,132,134,136,137,139,142,143,144,145,146,147]
Technology advancement	36/141	[56,57,58,63,69,71,74,77,85,90,94,95,109,116,119,120,121,123,124,125,126,127,128,129,130,131,132,133,134,135,136,137,138,139,140,141]
Sustainable technology advancement	27/36	[56,57,63,69,71,74,77,85,94,95,109,119,121,124,127,128,129,130,131,132,133,134,135,136,137,138,139]
High-yield seed varieties	8/36	[57,94,116,119,120,126,130,131]
Investment in R&D (e.g., precision farming)	4/36	[121,123,130,137]
Information technology and IT advancement	3/36	[130,137,138]
The use of mobile applications	3/36	[58,90,130]
The use of nanotechnology in agriculture	2/36	[130,140]
The use of biotechnology in agriculture	2/36	[130,141]
The use of genetically modified (GM) crop.	2/36	[57,125]
Local production enhancement	18/141	[57,69,85,87,89,94,98,103,105,109,112,117,120,134,137,144,148,149]
Farm production diversity	9/141	[57,64,69,105,106,116,118,120,185]
Building farmers capacities (small scale farmers)	18/141	[56,73,94,104,105,111,114,116,117,118,119,120,121,131,137,180,184,185]
Employment programs for poor households’ representatives	2/141	[110,152]
Public programs to influence diets in a healthy manner	9/141	[69,85,110,144,151,156,163,167,182]
Geopolitical and political stability	7/141	[69,98,104,117,123,124,142]
Food safety and food safety policies	16/141	[61,64,69,103,105,111,112,129,149,153,154,155,156,157,158,159]
Reduction of yield volatility	7/141	[85,94,105,119,124,126,175]
Agriculture infrastructure	7/141	[64,114,116,118,142,146,175]
The integrative policies (nexus)	6/141	[56,73,133,139,172,173]
The proper measurement of food security dimensions	4/141	[123,181,182,183]
The country’s natural resources (cultivated agriculture area)	9/141	[85,105,119,124,137,145,162,163,176]
The proper communication among all stakeholders	11/141	[6,56,68,69,84,92,129,130,156,157,168]
Management of government food reserves	7/141	[64,104,112,117,118,124,136]
Collaboration among all supply chain stakeholders	4/141	[75,130,134,157]
Promotion of the consumer’s education about sustainable consumption and healthy diet	12/141	[52,60,67,69,86,133,144,151,163,165,166,167]
Effective gleaning process (increasing the food bank’s processing resources)	8/141	[70,72,74,80,84,92,142,170]
Food distribution infrastructure	6/141	[71,75,76,112,177,178]
Adjustment in the diet structure	3/141	[59,86,163]
Dietary standard policies	4/141	[69,151,163,174]
Urban agriculture policies	3/141	[56,147,148]
The government role	16/141	[67,75,84,86,100,109,116,117,119,121,137,138,147,150,151,152]
Government capital investment in agriculture	7/16	[100,109,116,117,119,121,147]
Government and public administration’s commitment in enhancing the operational process of food distribution	3/16	[75,84,150]
Government regulation for food businesses and households that produce food waste	2/16	[67,86]
Government support for the research that enhances the country food security level	1/16	[137]
Government vision and commitment to adopt RFID technology	1/16	[138]
Government commitment in policy development to prevent obesity	1/16	[151]
Government knowledge of the correlation between market price and sustain the food prices during crises	1/16	[152]
Customer engagement in designing the public policies	1/141	[158]
Trust in the public institutions	1/141	[166]

## Appendix B. Summary Table of Most-Recommended Policies

**The Policy**	**Occurrence**	**Article Citations**
Food security policies	59/141	[56,57,63,64,65,69,81,85,87,89,91,94,97,98,99,101,102,103,104,105,106,107,108,109,110,111,112,113,114,115,116,117,118,119,120,121,122,123,124,126,127,130,131,133,134,137,142,144,145,148,149,151,152,175,177,180,182,184,185]
Food consumption polices that offer safety net	24/59	[65,81,99,101,102,104,105,107,108,109,110,112,113,114,115,116,118,119,122,137,144,148,151,152,182]
Policies to enhance small-scale farmer performance and assets base such as loans, subsidies, access to information and knowledge sharing	16/59	[89,104,105,109,111,114,116,117,118,119,121,131,137,149,180,184]
Government input subsidy programs (input subsidy policy) that provide farmers with subsidies to investment in high-yielding technology (e.g., automation, fertilizers, high-yield seed)	14/59	[89,101,109,114,116,121,124,126,127,130,131,137,149,175]
Rural development policies to reduce yield volatility and improve the agriculture infrastructure (e.g., irrigation and water-saving technologies)	14/59	[69,85,87,101,106,114,118,124,130,137,142,145,149,175]
Capacity building policies (educational, training and technical support)	14/59	[91,94,103,105,116,117,121,123,127,131,137,144,148,184]
Policies supporting locally produced food	12/59	[57,69,87,89,98,103,105,112,120,134,148,149]
Education policies in general	8/59	[69,97,101,105,117,127,130,133]
Diversified agriculture production policies	6/59	[57,64,106,116,118,185]
Policies that impact the farm-level commodity pricing	5/59	[63,105,116,120,175]
Food stock policies which help in predicting global food production information	4/59	[56,64,124,126]
Establishing policies to increase farmer income	4/59	[104,105,109,119]
Buffer stock policies	1/59	[104]
Resource allocation policies (income taxes)	1/59	[97]
Trade policies	20/141	[94,95,101,103,111,112,119,123,129,136,141,146,148,149,152,157,161,164,178,180]
Establishing infrastructure development policies that target agriculture logistic infrastructure and improve the speed and quality of shipping logistics	8/20	[94,112,119,136,146,149,157,164]
State trading and private trade supporting policies	7/20	[101,103,112,129,148,149,180]
Removal of tariff and non-tariff barrier	7/20	[94,95,111,123,146,152,161]
Trade infrastructure development policies	4/20	[94,112,123,141]
Reliable marine connection and transportation logistics policies	2/20	[164,178]
Food waste polices	49/141	[6,19,51,52,56,57,58,60,61,62,63,64,65,66,67,68,69,70,71,72,73,74,75,76,77,79,80,81,82,83,84,85,86,87,88,91,92,93,94,103,130,138,144,150,160,167,168,170,177]
Information and education campaigns that spread awareness at households and public level	21/49	[52,58,60,62,64,65,66,67,68,69,71,73,82,85,86,87,88,91,93,130,144]
Food waste reduction policies	17/49	[52,57,63,64,65,67,68,70,71,76,77,79,81,82,84,160,167]
Smart (innovative) food packaging and labelling policies	9/50	[6,56,58,62,77,88,91,94,103]
Food banks, food sharing or food rescue policies	8/49	[70,72,74,80,83,92,150,170]
Positive sanctions such as financial rewards, Tax credits, federal and state funding, vouchers, fewer taxes	8/49	[51,58,60,67,72,77,86,91]
Information and knowledge sharing among supply chain stakeholders	6/49	[6,56,74,92,138,168]
Comprehensive food waste legislation	6/49	[6,58,62,63,65,79]
Negative sanction policies by imposing fines and taxes such as disposal taxes	6/49	[51,58,60,65,77,86]
Food waste recycling polices	5/49	[61,62,67,77,84]
Technology advancement (mobile applications)	2/49	[58,130]
Gleaning operations policies (provide tax incentives and governmental support)	2/49	[72,92]
Nudging tool (nudge people in forming sustainable consumption behaviour)	2/49	[52,86]
Policies for peak storage reduction such as incentives for stock holding	2/49	[85,177]
Food waste management policy	1/49	[75]
Food upcycling with regards to market segmentation based on age	1/49	[19]
Food loss policy	10/141	[62,65,68,79,82,84,88,89,94,95]
Policies promoting the quantification of food loss	3/10	[68,82,88]
Food surplus policies	11/141	[51,58,70,72,75,76,79,82,84,90,91]
Policies to regulate company’s liability of donating surplus food	5/11	[51,75,82,84,91]
Food policies that subsidize purchases of surplus food “ugly food” by controlling for prices and the attributes of surplus items	2/11	[58,76]
Food safety policies	22/141	[61,64,69,70,103,105,111,112,120,125,129,130,137,138,149,153,154,155,156,157,158,159]
Food safety standards	7/22	[69,111,112,120,155,158,159]
Safety throughout the food supply chain	3/22	[130,138,157]
Developing food safety training policies	1/22	[155]
Mandatory state registration for major types of food additives	1/22	[103]
Food quality and food hygiene compliance certifications	5/22	[69,103,105,154,155]
The integrative and coherent policies between food, water, and energy system nexus.	4/141	[56,133,172,173]
Water–food (WF) nexus approach.	1/141	[139]
Food–energy–sanitation nexus approach	1/141	[73]
Water quality policies	8/141	[69,71,118,124,130,133,137,139]
Common agricultural policy (CAP) that addresses sustainable agriculture	16/141	[56,57,64,89,109,111,118,119,132,142,143,149,161,172,184,186]
Green and blue infrastructure (GBI) policies	1/16	[172]
Common agricultural policy (CAP) hinders the sustainable intensification	1/141	[121]
The policies that promote consumer education on sustainable consumption and improving consumer status consciousness and knowledge of their purchases ecological impact	6/141	[60,69,133,144,163,165]
Environmental policies	18/141	[59,73,94,120,121,124,130,135,139,141,145,147,159,160,161,162,163,166]
Gas emission policies, such as greenhouse gas reduction policies	2/141	[135,161]
Policies to reduce climate change impact	4/141	[124,139,162,163]
The coordination of policies between climate change, poverty and food insecurity due to their strong interlinking	4/141	[124,141,162,163]
Efficiency in agriculture water use, irrigation systems	3/141	[59,124,139]
The investments in water-saving technologies	2/141	[124,139]
Policies to minimize the impacts of anthropogenic activities on urban soils and enhance the urban agriculture practices	2/141	[147,159]
Soil contamination of heavy metals (cadmium)	1/141	[159]
Optimization of the fertilizer use policy	6/141	[59,73,94,120,130,145]
Carbon tax policy (promotes green economy)	2/141	[121,160]
Aquaculture environmental policies	1/141	[166]
Bio-energy policies	2/141	[73,161]
Management of government food reserves	7/141	[64,104,112,117,118,124,136]
Policies that promote traceability across the whole supply chain	10/141	[56,69,103,128,129,130,137,138,168,178]
Import policies	16/141	[94,95,100,103,104,105,109,112,116,117,119,120,124,126,134,146]
Direct governmental financial assistance to local agricultural assistance	8/16	[94,100,109,116,117,119,124,134]
Sustaining local agricultural product prices compared to the imported products	7/16	[95,100,104,105,116,120,146]
Providing temporary tax benefits for agriculture investment	4/16	[100,109,116,124]
Import ban (substitution) policies	4/16	[103,112,120,146]
Direct budget subsidies	2/16	[100,146]
Subsidizing loan interest rates	2/16	[100,117]
Diversification of imported food origins strategy	1/16	[126]
Risk management policies	10/141	[94,117,118,137,138,139,145,154,155,157]
Food scandals	2/10	[154,155]
COVID-19	1/10	[117]
Programmed risk identification	1/10	[139]
Proactive policy measures to handle the flood crises	2/10	[118,145]
Early warning systems for natural disasters	3/10	[94,137,145]
Risk management throughout the food supply chain	3/10	[138,139,157]
Dietary standard policies	4/141	[69,151,163,174]
Urban agriculture policies	3/141	[56,147,148]
Food aid policies	2/141	[118,150]
Policies discussed by one author only		
Devising the right population policy in China	1/141	[85]
Flexible retail modernization policies	1/141	[158]
Policies that facilitate short-term migration	1/141	[187]
Policy to stimulate equitable economic growth through manufacturing and services	1/141	[95]
Sound research governance policies: to address the expected and unexpected complications of new technologies (nanotechnology)	1/141	[140]

## References

[1] Berry E.M., Dernini S., Burlingame B., Meybeck A., Conforti P. (2015). Food security and sustainability: Can one exist without the other?. Public Health Nutr..

[2] Garnett T., Appleby M.C., Balmford A., Bateman I.J., Benton T.G., Bloomer P., Burlingame B., Dawkins M., Dolan L., Fraser D. (2013). Sustainable intensification in agriculture: Premises and policies. Science.

[3] Manikas I., Sundarakani B., Anastasiadis F., Ali B. (2022). A Framework for Food Security via Resilient Agri-Food Supply Chains: The Case of UAE. Sustainability.

[4] Ver Ploeg M., Breneman V., Farrigan T., Hamrick K., Hopkins D., Kaufman P., Lin B.-H., Nord M., Smith T.A., Williams R. (2009). Access to Affordable and Nutritious Food: Measuring and Understanding Food Deserts and Their Consequences: Report to Congress.

[5] Alabi M.O., Ngwenyama O. (2022). Food security and disruptions of the global food supply chains during COVID-19: Building smarter food supply chains for post COVID-19 era. Br. Food J..

[6] Halloran A., Clement J., Kornum N., Bucatariu C., Magid J. (2014). Addressing food waste reduction in Denmark. Food Policy.

[7] Godfray H.C.J., Beddington J.R., Crute I.R., Haddad L., Lawrence D., Muir J.F., Pretty J., Robinson S., Thomas S.M., Toulmin C. (2010). Food security: The challenge of feeding 9 billion people. Science.

[8] Kumar D., Kalita P. (2017). Reducing postharvest losses during storage of grain crops to strengthen food security in developing countries. Foods.

[9] Lipinski B., Hanson C., Waite R., Searchinger T., Lomax J. (2013). Reducing food loss and waste. World Resour. Inst. Work. Pap..

[10] Waldron A., Garrity D., Malhi Y., Girardin C., Miller D.C., Seddon N. (2017). Agroforestry can enhance food security while meeting other sustainable development goals. Trop. Conserv. Sci..

[11] Fanzo J. (2015). Ethical issues for human nutrition in the context of global food security and sustainable development. Glob. Food Secur..

[12] Pachapur P.K., Pachapur V.L., Brar S.K., Galvez R., Le Bihan Y., Surampalli R.Y. (2020). Food security and sustainability. Sustain. Fundam. Appl..

[13] Barling D., Fanzo J. (2018). Advances in Food Security and Sustainability.

[14] Tanksale A., Jha J. (2015). Implementing national food security act in India: Issues and challenges. Br. Food J..

[15] Parry M., Evans A., Rosegrant M.W., Wheeler T. (2009). Climate Change and Hunger: Responding to the Challenge.

[16] Abbass K., Qasim M.Z., Song H., Murshed M., Mahmood H., Younis I. (2022). A review of the global climate change impacts, adaptation, and sustainable mitigation measures. Environ. Sci. Pollut. Res..

[17] Lang T., Barling D. (2012). Food security and food sustainability: Reformulating the debate. Geogr. J..

[18] Concern-Worldwide and Welthungerhilfe (2019). The Challenge of Hunger and Climate Change. Fourteenth Annual Publication of the Global Hunger Index (GHI). https://reliefweb.int/report/world/2019-global-hunger-index-challenge-hunger-and-climate-change.

[19] Zhang J., Ye H., Bhatt S., Jeong H., Deutsch J., Ayaz H., Suri R. (2020). Addressing food waste: How to position upcycled foods to different generations. J. Consum. Behav..

[20] Tsolakis N., Srai J. (2017). A System Dynamics approach to food security through smallholder farming in the UK. Chem. Eng. Trans..

[21] Guiné R.d.P.F., Pato M.L.d.J., Costa C.a.d., Costa D.d.V.T.A.d., Silva P.B.C.d., Martinho V.J.P.D. (2021). Food Security and Sustainability: Discussing the Four Pillars to Encompass Other Dimensions. Foods.

[22] Clapp J., Moseley W.G., Burlingame B., Termine P. (2021). The case for a six-dimensional food security framework. Food Policy.

[23] HLPE (2020). Food Security and Nutrition: Building a Global Narrative Towards 2030. https://www.fao.org/3/ca9731en/ca9731en.pdf.

[24] Sperling L., McGuire S. (2012). Fatal gaps in seed security strategy. Food Secur..

[25] Economist Intelligence Unit (2019). Global Food Security Index 2019.

[26] Page M.J., McKenzie J.E., Bossuyt P.M., Boutron I., Hoffmann T.C., Mulrow C.D., Shamseer L., Tetzlaff J.M., Akl E.A., Brennan S.E. (2021). The PRISMA 2020 statement: An updated guideline for reporting systematic reviews. Int. J. Surgery.

[27] Brocke J.v., Simons A., Niehaves B., Niehaves B., Reimer K., Plattfaut R., Cleven A. Reconstructing the giant: On the importance of rigour in documenting the literature search process. Proceedings of the European Conference on Information Systerms.

[28] Mittal A. (2009). The 2008 Food Price Crisis: Rethinking Food Security Policies.

[29] Vilar-Compte M., Sandoval-Olascoaga S., Bernal-Stuart A., Shimoga S., Vargas-Bustamante A. (2015). The impact of the 2008 financial crisis on food security and food expenditures in Mexico: A disproportionate effect on the vulnerable. Public Health Nutr..

[30] Headey D.D. (2013). The impact of the global food crisis on self-assessed food security. World Bank Econ. Rev..

[31] De Amorim W.S., Valduga I.B., Ribeiro J.M.P., Williamson V.G., Krauser G.E., Magtoto M.K., de Andrade J.B.S.O. (2018). The nexus between water, energy, and food in the context of the global risks: An analysis of the interactions between food, water, and energy security. Environ. Impact Assess. Rev..

[32] Hameed M., Ahmadalipour A., Moradkhani H. (2020). Drought and food security in the middle east: An analytical framework. Agric. For. Meteorol..

[33] Javier Arturo Orjuela C., Adarme J.W. (2017). Dynamic impact of the structure of the supply chain of perishable foods on logistics performance and food security. J. Ind. Eng. Manag..

[34] Murti Mulyo Aji J. (2020). Linking Supply Chain Management and Food Security: A Concept of Building Sustainable Competitive Advantage of Agribusiness in Developing Economies. E3S Web Conf..

[35] Irani Z., Sharif A.M., Lee H., Aktas E., Topaloğlu Z., van’t Wout T., Huda S. (2018). Managing food security through food waste and loss: Small data to big data. Comput. Oper. Res..

[36] Katajajuuri J.M., Silvennoinen K., Hartikainen H., Heikkilä L., Reinikainen A. (2014). Food waste in the Finnish food chain. J. Clean. Prod..

[37] Cooper M.C., Ellram L.M. (1993). Characteristics of supply chain management and the implications for purchasing and logistics strategy. Int. J. Logist. Manag..

[38] Stone J., Rahimifard S. (2018). Resilience in agri-food supply chains: A critical analysis of the literature and synthesis of a novel framework. Supply Chain. Manag..

[39] Namany S., Govindan R., Alfagih L., McKay G., Al-Ansari T. (2020). Sustainable food security decision-making: An agent-based modelling approach. J. Clean. Prod..

[40] Barrett C.B. (2010). Measuring food insecurity. Science.

[41] Rosegrant M.W., Cline S.A. (2003). Global food security: Challenges and policies. Science.

[42] Philippidis G., Sartori M., Ferrari E., M’Barek R. (2019). Waste not, want not: A bio-economic impact assessment of household food waste reductions in the EU. Resour. Conserv. Recycl..

[43] Basher S.A., Raboy D., Kaitibie S., Hossain I. (2013). Understanding challenges to food security in dry Arab micro-states: Evidence from Qatari micro-data. J. Agric. Food Ind. Organ..

[44] Cattaneo A., Sánchez M.V., Torero M., Vos R. (2020). Reducing food loss and waste: Five challenges for policy and research. Food Policy.

[45] Peter Timmer C., Dawe D. (2007). Managing food price instability in Asia: A macro food security perspective. Asian Econ. J..

[46] Timmer C.P. (2015). Food Security and Scarcity: Why Ending Hunger Is So Hard.

[47] Chand R. (2007). International trade, food security, and the response to the WTO in South Asian Countries. Food Secur. Indic. Meas. Impact Trade Openness.

[48] Pizzi S., Caputo A., Corvino A., Venturelli A. (2020). Management research and the UN sustainable development goals (SDGs): A bibliometric investigation and systematic review. J. Clean. Prod..

[49] Light R.J., Pillemer D.B. (1984). Summing Up: The Science of Reviewing Research.

[50] Zhou X., Jin Y., Zhang H., Li S., Huang X. A map of threats to validity of systematic literature reviews in software engineering. Proceedings of the Asia-Pacific Software Engineering Conference (APSEC).

[51] Aamir M., Ahmad H., Javaid Q., Hasan S.M. (2018). Waste Not, Want Not: A Case Study on Food Waste in Restaurants of Lahore, Pakistan. J. Food Prod. Mark..

[52] Von Kameke C., Fischer D. (2018). Preventing household food waste via nudging: An exploration of consumer perceptions. J. Clean. Prod..

[53] Aktas E., Sahin H., Topaloglu Z., Oledinma A., Huda A.K.S., Irani Z., Sharif A.M., van’t Wout T., Kamrava M. (2018). A consumer behavioural approach to food waste. J. Enterp. Inf. Manag..

[54] Boschini M., Falasconi L., Giordano C., Alboni F. (2018). Food waste in school canteens: A reference methodology for large-scale studies. J. Clean. Prod..

[55] Ellison B., Lusk J.L. (2018). Examining Household Food Waste Decisions: A Vignette Approach. Appl. Econ. Perspect. Policy.

[56] Irani Z., Sharif A.M. (2016). Sustainable food security futures: Perspectives on food waste and information across the food supply chain. J. Enterp. Inf. Manag..

[57] Notarnicola B., Hayashi K., Curran M.A., Huisingh D. (2012). Progress in working towards a more sustainable agri-food industry. J. Clean. Prod..

[58] Schanes K., Dobernig K., Gözet B. (2018). Food waste matters—A systematic review of household food waste practices and their policy implications. J. Clean. Prod..

[59] Tian X., Engel B.A., Qian H., Hua E., Sun S., Wang Y. (2021). Will reaching the maximum achievable yield potential meet future global food demand?. J. Clean. Prod..

[60] Zorpas A.A., Lasaridi K., Pociovalisteanu D.M., Loizia P. (2018). Monitoring and evaluation of prevention activities regarding household organics waste from insular communities. J. Clean. Prod..

[61] Ermgassen E., Phalan B., Green R.E., Balmford A. (2016). Reducing the land use of EU pork production: Where there’s swill, there’s a way. Food Policy.

[62] Garcia-Herrero I., Hoehn D., Margallo M., Laso J., Bala A., Batlle-Bayer L., Fullana P., Vazquez-Rowe I., Gonzalez M.J., Dura M.J. (2018). On the estimation of potential food waste reduction to support sustainable production and consumption policies. Food Policy.

[63] Jafari Y., Britz W., Dudu H., Roson R., Sartori M. (2020). Can food waste reduction in Europe help to increase food availability and reduce pressure on natural resources globally?. Ger. J. Agric. Econ..

[64] Kwasek M. (2012). Threats to food security and Common agricultural policy. Ekon. Poljopr. -Econ. Agric..

[65] Katare B., Serebrennikov D., Wang H.H., Wetzstein M. (2017). Social-Optimal Household Food Waste: Taxes and Government Incentives. Am. J. Agric. Econ..

[66] Septianto F., Kemper J.A., Northey G. (2020). Thanks, but no thanks: The influence of gratitude on consumer awareness of food waste. J. Clean. Prod..

[67] Warshawsky D.N. (2015). The devolution of urban food waste governance: Case study of food rescue in Los Angeles. Cities.

[68] Koester U. (2015). Reduction of Food Loss and Waste: An Exaggerated Agitation. Eurochoices.

[69] Mayton H., Beal T., Rubin J., Sanchez A., Heller M., Hoey L., de Haan S., Duong T.T., Huynh T., Burra D.D. (2020). Conceptualizing sustainable diets in Vietnam: Minimum metrics and potential leverage points. Food Policy.

[70] Lindberg R., Lawrence M., Gold L., Friel S. (2014). Food rescue—An australian example. Br. Food J..

[71] Ridoutt B.G., Juliano P., Sanguansri P., Sellahewa J. (2010). The water footprint of food waste: Case study of fresh mango in Australia. J. Clean. Prod..

[72] Lee D.S., Sonmez E., Gomez M.I., Fan X.L. (2017). Combining two wrongs to make two rights: Mitigating food insecurity and food waste through gleaning operations. Food Policy.

[73] Tucho G.T., Okoth T. (2020). Evaluation of neglected bio-wastes potential with food-energy-sanitation nexus. J. Clean. Prod..

[74] Nica-Avram G., Harvey J., Smith G., Smith A., Goulding J. (2020). Identifying food insecurity in food sharing networks via machine learning. J. Bus. Res..

[75] Thapa Karki S., Bennett A.C.T., Mishra J.L. (2020). Reducing food waste and food insecurity in the UK: The architecture of surplus food distribution supply chain in addressing the sustainable development goals (Goal 2 and Goal 12.3GG) at a city level. Ind. Mark. Manag..

[76] Richards T.J., Hamilton S.F. (2018). Food waste in the sharing economy. Food Policy.

[77] Krishnan R., Agarwal R., Bajada C., Arshinder K. (2020). Redesigning a food supply chain for environmental sustainability—An analysis of resource use and recovery. J. Clean. Prod..

[78] Song G., Semakula H.M., Fullana-i-Palmer P. (2018). Chinese household food waste and its’ climatic burden driven by urbanization: A Bayesian Belief Network modelling for reduction possibilities in the context of global efforts. J. Clean. Prod..

[79] Kotykova O., Babych M. (2019). Limitations in availability of food in Ukraine as a result of loss and waste. Oeconomia Copernic..

[80] Gitler J., Kroch G., Bellinsky J., Fiedler D. (2017). Social Enterprise Business Sustainability of the Food Banking Model the Case of Leket Israel, Israel’s National Food Bank. Bus. Peace Sustain. Dev..

[81] Hamilton S.F., Richards T.J. (2019). Food policy and household food waste. Am. J. Agric. Econ..

[82] Khalid S., Naseer A., Shahid M., Shah G.M., Ullah M.I., Waqar A., Abbas T., Imran M., Rehman F. (2019). Assessment of nutritional loss with food waste and factors governing this waste at household level in Pakistan. J. Clean. Prod..

[83] Morone P., Falcone P.M., Imbert E., Morone A. (2018). Does food sharing lead to food waste reduction? An experimental analysis to assess challenges and opportunities of a new consumption model. J. Clean. Prod..

[84] Sambo N., Hlengwa D.C. (2018). Post-flight food waste and corporate social responsibility at South Africa Airways: Perceptions of employees at Air Chefs South Africa. Afr. J. Hosp. Tour. Leis..

[85] Yuneng D., Youliang X., Leiyong Z., Leiyong Z., Shufang S. (2020). Can China’s food production capability meet her peak food demand in the future?. Int. Food Agribus. Manag. Rev..

[86] Xu Z.G., Zhang Z.L., Liu H.Y., Zhong F.N., Bai J.F., Cheng S.K. (2020). Food-away-from-home plate waste in China: Preference for variety and quantity. Food Policy.

[87] Abbade E.B. (2020). Estimating the nutritional loss and the feeding potential derived from food losses worldwide. World Dev..

[88] Buzby J.C., Hyman J. (2012). Total and per capita value of food loss in the United States. Food Policy.

[89] Feukam Nzudie H.L., Zhao X., Liu G., Tillotson M.R., Hou S., Li Y. (2021). Driving force analysis for food loss changes in Cameroon. J. Clean. Prod..

[90] Apostolidis C., Brown D., Wijetunga D., Kathriarachchi E. (2021). Sustainable value co-creation at the Bottom of the Pyramid: Using mobile applications to reduce food waste and improve food security. J. Mark. Manag..

[91] Baglioni S., de Pieri B., Tallarico T. (2017). Surplus Food Recovery and Food Aid: The Pivotal Role of Non-profit Organisations. Insights Italy Germany. Volunt..

[92] Sonmez E., Lee D., Gomez M.I., Fan X.L. (2016). Improving food bank gleaning operations: An application in new york state. Am. J. Agric. Econ..

[93] Landry C., Smith T.A., Turner D. (2018). Food waste and food retail density. J. Food Prod. Mark..

[94] Popat M., Griffith G., Mounter S., Cacho O. (2020). Postharvest losses at the farm level and its economy-wide costs: The case of the maize sector in Mozambique. Agrekon.

[95] Rutten M., Kavallari A. (2016). Reducing food losses to protect domestic food security in the Middle East and North Africa. Afr. J. Agric. Resour. Econ. -Afjare.

[96] Gustavsson J., Cederberg C., Sonesson U., van Otterdijk R., Meybeck A. (2011). Global Food Losses and Food Waste: Extent. Causes and Prevention.

[97] Abdelhedi I.T., Zouari S.Z. (2020). Agriculture and food security in North Africa: A theoretical and empirical approach. J. Knowl. Econ..

[98] Ahmed J.U., Begum F., Ahmed A., Talukder N. (2020). A blessing inside a calamity: Baladna food industries in Qatar. Int. J. Manag. Enterp. Dev..

[99] Beghin J., Meade B., Rosen S. (2017). A food demand framework for International Food Security Assessment. J. Policy Model..

[100] Boldyreva I., Andryushchenko O., Nikitaeva A., Udalova Z., Rudash J. (2017). The agricultural production and food industry development trends in the context of food security of Russia. J. Environ. Manag. Tour..

[101] Boratynska K., Huseynov R.T. (2017). An innovative approach to food security policy in developing countries. J. Innov. Knowl..

[102] Debnath D., Babu S., Ghosh P., Helmar M. (2018). The impact of India’s food security policy on domestic and international rice market. J. Policy Model..

[103] Gnezdova J.V., Barilenko V.I., Kozenkova T.A., Chernyshev A.V., Vasina N.V. (2018). Food safety auditing in Russia in a climate of foreign sanctions and a policy of import substitution. Qual.—Access Success.

[104] Jamaludin M., Fauzi T.H., Nugraha D.N.S., Adnani L. (2020). Service supply chain management in the performance of national logistics agency in national food security. Int. J. Supply Chain. Manag..

[105] Jouzi Z., Azadi H., Taheri F., Zarafshani K., Gebrehiwot K., Van Passel S., Lebailly P. (2017). Organic Farming and Small-Scale Farmers: Main Opportunities and Challenges. Ecol. Econ..

[106] Marivoet W., Ulimwengu J., Sedano F. (2019). Spatial typology for targeted food and nutrition security interventions. World Dev..

[107] Kishore A., Chakrabarti S. (2015). Is more inclusive more effective? The ‘New Style’ public distribution system in India. Food Policy.

[108] Lentz E.C., Barrett C.B. (2013). The economics and nutritional impacts of food assistance policies and programs. Food Policy.

[109] Kormishkina L.A., Semenova N.N. (2016). Monitoring of food security in the Russian Federation: Methodology and assessment. Eur. Res. Stud. J..

[110] Mahadevan R., Suardi S. (2014). Regional Differences Pose Challenges for Food Security Policy: A Case Study of India. Reg. Stud..

[111] Maggio A., van Criekinge T., Malingreau J. (2016). Global food security: Assessing trends in view of guiding future EU policies. Foresight.

[112] Melnikov A.B., Mikhailushkin P.V., Poltarykhin A.L., Dibrova Z.N. (2019). Economic aspects of the resolution of the issue of food security: A case study. Entrep. Sustain. Issues.

[113] Miller C.M., Tsoka M., Reichert K. (2011). The impact of the Social Cash Transfer Scheme on food security in Malawi. Food Policy.

[114] Mockshell J., Birner R. (2020). Who has the better story? On the narrative foundations of agricultural development dichotomies. World Dev..

[115] Munesue Y., Masui T., Fushima T. (2015). The effects of reducing food losses and food waste on global food insecurity, natural resources, and greenhouse gas emissions. Environ. Econ. Policy Stud..

[116] Mumin Y.A., Abdulai A. (2020). Informing Food Security and Nutrition Strategies in Sub-Saharan African Countries: An Overview and Empirical AnalysisJEL codes. Appl. Econ. Perspect. Policy.

[117] Omodero C.O., Adetula D.T., Iyoha F.O., Odianonsen F. (2020). Agricultural revamping via major capital outlay: The antidote to food insecurity challenges in Nigeria. Acad. Entrep. J..

[118] Oskorouchi H.R., Sousa-Poza A. (2021). Floods, food security, and coping strategies: Evidence from Afghanistan. Agric. Econ..

[119] Pan J., Chen Y., Zhang Y., Chen M., Fennell S., Luan B., Wang F., Meng D., Liu Y., Jiao L. (2020). Spatial-temporal dynamics of grain yield and the potential driving factors at the county level in China. J. Clean. Prod..

[120] Polimeni J.M., Iorgulescu R.I., Bǎlan M. (2014). Organic farming, bridge between food security and food safety. Qual. Access Success.

[121] Rickard S. (2015). Food Security and Climate Change: The Role of Sustainable Intensification, the Importance of Scale and the CAP. Eurochoices.

[122] Schmidt L., Shore-Sheppard L., Watson T. (2016). The effect of safety-net programs on food insecurity. J. Hum. Resour..

[123] Stezhko N. (2016). Global indices in assessment of the global food problem and its impact factor. Econ. Ann.-XXI.

[124] Wiebelt M., Breisinger C., Ecker O., Al-Riffai P., Robertson R., Thiele R. (2013). Compounding food and income insecurity in Yemen: Challenges from climate change. Food Policy.

[125] Adenle A.A., Morris E.J., Parayil G. (2013). Status of development, regulation and adoption of GM agriculture in Africa: Views and positions of stakeholder groups. Food Policy.

[126] Araujo-Enciso S.R., Fellmann T. (2020). Yield Variability and Harvest Failures in Russia, Ukraine and Kazakhstan and Their Possible Impact on Food Security in the Middle East and North Africa. J. Agric. Econ..

[127] Devkota K.P., Khanda C.M., Beebout S.J., Mohapatra B.K., Singleton G.R., Puskur R. (2020). Assessing alternative crop establishment methods with a sustainability lens in rice production systems of Eastern India. J. Clean. Prod..

[128] Dinesh Kumar K., Kumar D.S.M., Anandh R. (2020). Blockchain technology in food supply chain security. Int. J. Sci. Technol. Res..

[129] Ene C. (2013). Food Traceability—Actual Coordinates at National, European and International Level. Qual.—Access Success.

[130] Shukla S., Singh S., Shankar R. (2015). Food security and technology in India. Int. J. Sustain. Agric. Manag. Inform..

[131] Sinyolo S. (2020). Technology adoption and household food security among rural households in South Africa: The role of improved maize varieties. Technol. Soc..

[132] Li L., Chong C., Wang C.H., Wang X. (2020). A decision support framework for the design and operation of sustainable urban farming systems. J. Clean. Prod..

[133] Niva V., Cai J., Taka M., Kummu M., Varis O. (2020). China’s sustainable water-energy-food nexus by 2030: Impacts of urbanization on sectoral water demand. J. Clean. Prod..

[134] Margunani M., Melati I.S., Soesilowati E. (2018). A modelling framework of sustainable supply chain management for organic vegetables in rural area with narrow land: An action research in Indonesia. Int. J. Supply Chain. Manag..

[135] Rohmer S.U.K., JGerdessen C., Claassen G.D.H. (2019). Sustainable supply chain design in the food system with dietary considerations: A multi-objective analysis. Eur. J. Oper. Res..

[136] Stehnei M., Irtyshcheva I., Boiko Y., Rogatina L., Khaustova K. (2018). Conceptual approaches to the formation of regional food security strategy in the context of sustainable development. Probl. Perspect. Manag..

[137] Tokhayeva Z.O., Almukhambetova B.Z., Keneshbayev B., Akhmetova K. (2020). Innovative processes’ management in agriculture and food security: Development opportunities. Entrep. Sustain. Issues.

[138] Wen X.W., Marlin J., Wen Z.J., Yang Z.H. (2020). Reviewing studies of radio frequency identification applications in supply chain for food safety. Int. Food Agribus. Manag. Rev..

[139] Zeng X., Zhao J., Wang D., Kong X., Zhu Y., Liu Z., Dai W., Huang G. (2019). Scenario analysis of a sustainable water-food nexus optimization with consideration of population-economy regulation in Beijing-Tianjin-Hebei region. J. Clean. Prod..

[140] Sastry R.K., Rashmi H.B., Rao N.H. (2011). Nanotechnology for enhancing food security in India. Food Policy.

[141] Wu F., Wang Y., Liu Y., Liu Y., Zhang Y. (2021). Simulated responses of global rice trade to variations in yield under climate change: Evidence from main rice-producing countries. J. Clean. Prod..

[142] Bazgǎ B., Rebega L. (2014). Sustainable development—Theoretical power dimensions of food security. Qual. Access Success.

[143] Boboc D., Constantin F., Diaconeasa M.C. (2019). The use of sustainability concept regarding dairy and fruits in the web of science papers. Qual. Access Success.

[144] Matzembacher D.E., Meira F.B. (2019). Sustainability as business strategy in community supported agriculture: Social, environmental and economic benefits for producers and consumers. Br. Food J..

[145] Qi X., Vitousek P.M., Liu L. (2015). Identification and evaluation of risk factors related to provincial food insecurity in China. J. Risk Res..

[146] Poltarykhin A.L., Suray N.M., Zemskov Y.V., Abramov Y.V., Glotko A.V. (2018). Food safety in the Russian Federation, its problems with the solutions. Acad. Strateg. Manag. J..

[147] Salomon M.J., Watts-Williams S.J., McLaughlin M.J., Cavagnaro T.R. (2020). Urban soil health: A city-wide survey of chemical and biological properties of urban agriculture soils. J. Clean. Prod..

[148] Dieleman H. (2017). Urban agriculture in Mexico City; balancing between ecological, economic, social and symbolic value. J. Clean. Prod..

[149] Bandoi A., Bocean C., Florea A., Simion D., Sitnikov C. (2019). The perspective of cluster ranking analysis in the development of food safety measures. Amfiteatru Econ..

[150] Rovati G. (2017). The contribution of food banks to social solidarity and positive peace the Italian case. Bus. Peace Sustain. Dev..

[151] Sisnowski J., Street J.M., Braunack-Mayer A. (2016). Targeting population nutrition through municipal health and food policy: Implications of New York City’s experiences in regulatory obesity prevention. Food Policy.

[152] Van Wyk R.B., Dlamini C.S. (2018). The impact of food prices on the welfare of households in South Africa. S. Afr. J. Econ. Manag. Sci..

[153] Alieva N.M. (2017). The food quality and safety as indicator of the national food safety assessment. Qual. Access Success.

[154] Li Y.F., Phau I., Lu W., Teah M. (2018). Crisis management of food security scandals in China: Motivations and solutions towards purchase intention. J. Consum. Behav..

[155] Leong J.K., Hancer M. (2014). Crisis management preparedness to protect food products in a foodservice operation. J. Hosp. Mark. Manag..

[156] Mylona K., Maragkoudakis P., Miko L., Bock A.K., Wollgast J., Caldeira S., Ulberth F. (2018). Viewpoint: Future of food safety and nutrition—Seeking win-wins, coping with trade-offs. Food Policy.

[157] Septiani W., Marimin M., Herdiyeni Y., Haditjaroko L. (2016). Method and approach mapping for agri-food supply chain risk management: A literature review. Int. J. Supply Chain. Manag..

[158] Wertheim-Heck S.C.O., Vellema S., Spaargaren G. (2015). Food safety and urban food markets in Vietnam: The need for flexible and customized retail modernization policies. Food Policy.

[159] Zhan J., Twardowska I., Wang S., Wei S., Chen Y., Ljupco M. (2019). Prospective sustainable production of safe food for growing population based on the soybean (Glycine max L. Merr.) crops under Cd soil contamination stress. J. Clean. Prod..

[160] Ntombela S.M., Bohlmann H.R., Kalaba M.W. (2019). Greening the South Africa’s Economy Could Benefit the Food Sector: Evidence from a Carbon Tax Policy Assessment. Environ. Resour. Econ..

[161] Philippidis G., Bartelings H., Smeets E. (2018). Sailing into Unchartered Waters: Plotting a Course for EU Bio-Based Sectors. Ecol. Econ..

[162] Sassi M., Cardaci A. (2013). Impact of rainfall pattern on cereal market and food security in Sudan: Stochastic approach and CGE model. Food Policy.

[163] Von Ow A., Waldvogel T., Nemecek T. (2020). Environmental optimization of the Swiss population’s diet using domestic production resources. J. Clean. Prod..

[164] Gani A. (2020). Achieving food security through live animal imports in the Gulf Cooperation Council countries. Br. Food J..

[165] Marx-Pienaar N.J.M.M., Erasmus A.C. (2014). Status consciousness and knowledge as potential impediments of households’ sustainable consumption practices of fresh produce amidst times of climate change. Int. J. Consum. Stud..

[166] Oliva R.D.P., Vasquez-Lavin F., San Martin V.A., Hernandez J.I., Vargas C.A., Gonzalez P.S., Gelcich S. (2019). Ocean Acidification, Consumers’ Preferences, and Market Adaptation Strategies in the Mussel Aquaculture Industry. Ecol. Econ..

[167] Yu Y., Jaenicke E.C. (2020). Estimating food waste as household production inefficiency. Am. J. Agric. Econ..

[168] Kayikci Y., Subramanian N., Dora M., Bhatia M.S. (2020). Food supply chain in the era of Industry 4.0: Blockchain technology implementation opportunities and impediments from the perspective of people, process, performance, and technology. Prod. Plan. Control..

[169] Iese V., Paeniu L., Pouvalu S., Tuisavusavu A., Bosenaqali S., Wairiu M., Devi A. (2015). Food Security: Best Practices from the Pacific. Pacific Centre for Environment and Sustainable Development (PaCE-SD).

[170] Mirosa M., Mainvil L., Horne H., Mangan-Walker E. (2016). The social value of rescuing food, nourishing communities. Br. Food J..

[171] Bruins H.J., Bu F. (2006). Food security in China and contingency planning: The significance of grain reserves. J. Contingencies Crisis Manag..

[172] Bellezoni R.A., Meng F., He P., Seto K.C. (2021). Understanding and conceptualizing how urban green and blue infrastructure affects the food, water, and energy nexus: A synthesis of the literature. J. Clean. Prod..

[173] Karabulut A.A., Crenna E., Sala S., Udias A. (2018). A proposal for integration of the ecosystem-water-food-land-energy (EWFLE) nexus concept into life cycle assessment: A synthesis matrix system for food security. J. Clean. Prod..

[174] Luan Y., Fischer G., Wada Y., Sun L., Shi P. (2018). Quantifying the impact of diet quality on hunger and undernutrition. J. Clean. Prod..

[175] Arata L., Fabrizi E., Sckokai A. (2020). worldwide analysis of trend in crop yields and yield variability: Evidence from FAO data. Econ. Model..

[176] Dobre I., Davidescu A.A., Apostu S.A. (2020). Analysing food security at European level. Econ. Comput. Econ. Cybern. Stud. Res..

[177] Singha Mahapatra M., Mahanty B. (2020). Policies for managing peak stock of food grains for effective distribution: A case of the Indian food program. Socio-Econ. Plan. Sci..

[178] Stafiychuk I., Kutliyarov A., Kutliyarov D., Galeev E., Lukmanova A., Gubaydullina G. (2019). Specific aspects of land use planning and forecasting for effective supply chain management. Int. J. Supply Chain. Manag..

[179] Bagnied M.A., Speece M. (2019). Marketing and regional integration for food security in the arab world. J. Macromark..

[180] Montalbano P., Pietrelli R., Salvatici L. (2018). Participation in the market chain and food security: The case of the Ugandan maize farmers. Food Policy.

[181] Bertelli O. (2020). Food security measures in sub-saharan Africa. a validation of the lsms-isa scale. J. Afr. Econ..

[182] Robe M., Procopie R., Bucur M. (2019). Exploring the role of individual food security in the assessment of population’s food safety. Amfiteatru Econ..

[183] Sedikova I., Savenko I., Boiko O. (2018). Food security of the black sea littoral and features of its development. Balt. J. Econ. Stud..

[184] Impiglia A., Lewis P. (2019). Combatting food insecurity and rural poverty through enhancing small-scale family farming in the Near East and North Africa. New Medit.

[185] Akerele D., Shittu A.M. (2017). Can food production diversity influence farm households’ dietary diversity? An appraisal from two-dimensional food diversity measures. Int. J. Soc. Econ..

[186] Bala B.K., Bhuiyan M.G.K., Alam M.M., Arshad F.M., Sidique S.F., Alias E.F. (2017). Modelling of supply chain of rice in Bangladesh. Int. J. Syst. Sci. Oper. Logist..

[187] Minh C.N., Winters P. (2011). The impact of migration on food consumption patterns: The case of Vietnam. Food Policy.

[188] Kemmerling B., Schetter C., Wirkus L. (2022). The logics of war and food (in) security. Glob. Food Secur..

[189] Abiad M.G., Meho L.I. (2018). Food loss and food waste research in the Arab world: A systematic review. Food Secur..

[190] Ribeiro J.M.P., Berchin I.I., da Silva Neiva S., Soares T., de Albuquerque Junior C.L., Deggau A.B., de Amorim W.S., Barbosa S.B., Secchi L., de Andrade Guerra J.B.S.O. (2021). Food stability model: A framework to support decision-making in a context of climate change. Sustain. Dev..

[191] Shafiee-Jood M., Cai X. (2016). Reducing food loss and waste to enhance food security and environmental sustainability. Environ. Sci. Technol..

[192] Read Q.D., Brown S., Cuéllar A.D., Finn S.M., Gephart J.A., Marston L.T., Meyer E., Weitz K.A., Muth M.K. (2020). Assessing the environmental impacts of halving food loss and waste along the food supply chain. Sci. Total Environ..

[193] Jalava M., Guillaume J.H., Kummu M., Porkka M., Siebert S., Varis O. (2016). Diet change and food loss reduction: What is their combined impact on global water use and scarcity?. Earth’s Future.

[194] Lu S., Cheng G., Li T., Xue L., Liu X., Huan J., Liu G. (2022). Quantifying supply chain food loss in China with primary data: A large-scale, field-survey based analysis for staple food, vegetables, and fruits. Resour. Conserv. Recycl..

[195] Kummu M., De Moel H., Porkka M., Siebert S., Varis O., Ward P.J. (2012). Lost food, wasted resources: Global food supply chain losses and their impacts on freshwater, cropland, and fertiliser use. Sci. Total Environ..

[196] Neumann K., Verburg P.H., Stehfest E., Müller C. (2010). The yield gap of global grain production: A spatial analysis. Agric. Syst..

[197] Chen C., Chaudhary A., Mathys A. (2020). Nutritional and environmental losses embedded in global food waste. Resour. Conserv. Recycl..

[198] Shabanali Fami H., Aramyan L.H., Sijtsema S.J., Alambaigi A. (2021). The relationship between household food waste and food security in Tehran city: The role of urban women in household management. Ind. Mark. Manag..

[199] Hughes S., Zhai J., Hallstrom J.O. Waste auditing sensor technology to enhance the reduction of edible discards in university cafeterias & eateries. Proceedings of the 2018 IEEE International Conference on Smart Computing (SMARTCOMP).

